# A scalable machine learning approach for measuring violent and peaceful forms of political protest participation with social media data

**DOI:** 10.1371/journal.pone.0212834

**Published:** 2019-03-19

**Authors:** Lefteris Jason Anastasopoulos, Jake Ryland Williams

**Affiliations:** 1 Department of Public Administration and Policy, School of Public and International Affairs, University of Georgia, Athens, Georgia, United States of America; 2 Department of Political Science, School of Public and International Affairs, University of Georgia, Athens, Georgia, United States of America; 3 Institute for Artificial Intelligence, University of Georgia, Athens, Georgia, United States of America; 4 College of Computing and Informatics. Drexel University, Philadelphia, Pennsylvania, United States of America; Rutgers The State University of New Jersey, UNITED STATES

## Abstract

In this paper, we introduce a scalable machine learning approach accompanied by open-source software for identifying violent and peaceful forms of political protest participation using social media data. While violent political protests are statistically rare events, they often shape public perceptions of political and social movements. This is, in part, due to the extensive and disproportionate media coverage which violent protest participation receives relative to peaceful protest participation. In the past, when a small number of media conglomerates served as the primary information source for learning about political and social movements, viewership and advertiser demands encouraged news organizations to focus on violent forms of political protest participation. Consequently, much of our knowledge about political protest participation is derived from data collected about violent protests, while less is known about peaceful forms of protest. Since the early 2000s, the digital revolution shifted attention away from traditional news sources toward social media as a primary source of information about current events. This, along with developments in machine learning which allow us to collect and analyze data relevant to political participation, present us with unique opportunities to expand our knowledge of peaceful and violent forms of political protest participation through social media data.

## Introduction

While violent protests are statistically rare events, they tend to shape how political and social movements are perceived by the public [1, 2]. This is likely due to the fact that violent protests receive far more media coverage than peaceful protests addressing similar causes [3, 4]. Smith et al. (2001) for example, compare police records of registered protests with actual coverage of protests and find that only a very small proportion of registered protests are covered by news media, and those protests which tended to be covered often involved violence. Similarly, using a content analysis of major newspapers covering the Global Justice movement which occurred between 1999 and 2000, Boycoff (2006) finds that violent acts committed by Global Justice protesters were heavily emphasized in news reports of the movement.

As a result of the focus on violent protests, much of our prior knowledge about political protest participation is based on data collected about violent protest activity while less is known about participation in peaceful protest [5]. Since the early 2000s, however, the digital revolution ushered in a shift from reliance on major media conglomerates as a primary source of news information to social media. The shift in attention, along with developments in machine learning techniques which provide a means of collecting and analyzing text posts about protest activity, present a unique opportunity to expand our knowledge about political protest participation using data from both peaceful and violent forms of political protest participation.

In this paper, we seek to develop a practical methodology, accompanied by open—source software, which allows researchers and the public to build databases that can identify and measure participation in peaceful and violent political protest events from social media data. Here, we introduce a scalable machine learning approach and software for identifying individual and group participation in violent and peaceful forms of protest participation using social media data. By leveraging spatial and textual data to identify violent and peaceful forms of political protest participation using English language Tweets, we demonstrate how our methodology and our software can be used by researchers to construct rich databases which measure participation in violent and peaceful forms of political protest activity at fine-grained levels of time and geography.

## Social media and political participation

In recent years, social media has become a primary means of political protest participation and has been identified as a legitimate form of political protest participation by scholars studying these phenomena [6]. Recent evidence points to the conclusion that social media was instrumental for on the ground mobilization efforts for the Arab Spring [7], Occupy Wall Street and Black Lives Matter movements and accompanying citizen demonstrations [8, 9]. Because massive quantities of social media data are publicly available on the Internet, adoption of this relatively new technology for mobilization purposes provides researchers with an unprecedented opportunity to learn about political protest participation from these data. Accomplishing this, however, requires identifying a proverbial needle in a haystack.

While massive amounts of social media data are *available* to the public at little to no cost, utilizing this data to learn about political protest participation requires developing a systematic means of distinguishing between data relevant to different forms of political protest participation and data that are irrelevant to these activities from conceptual and a technical standpoint. Conceptually, a method which can distinguish between data relevant to political participation must first determine how activities related to these phenomena manifest within social media data, for example, as tweets on Twitter discussing participation in a citizen demonstration or expressing a political viewpoint about an issue. This knowledge can then be utilized to develop a classification scheme can be used to assign labels to individual pieces of social media data such as tweets.

**Fig 1:**
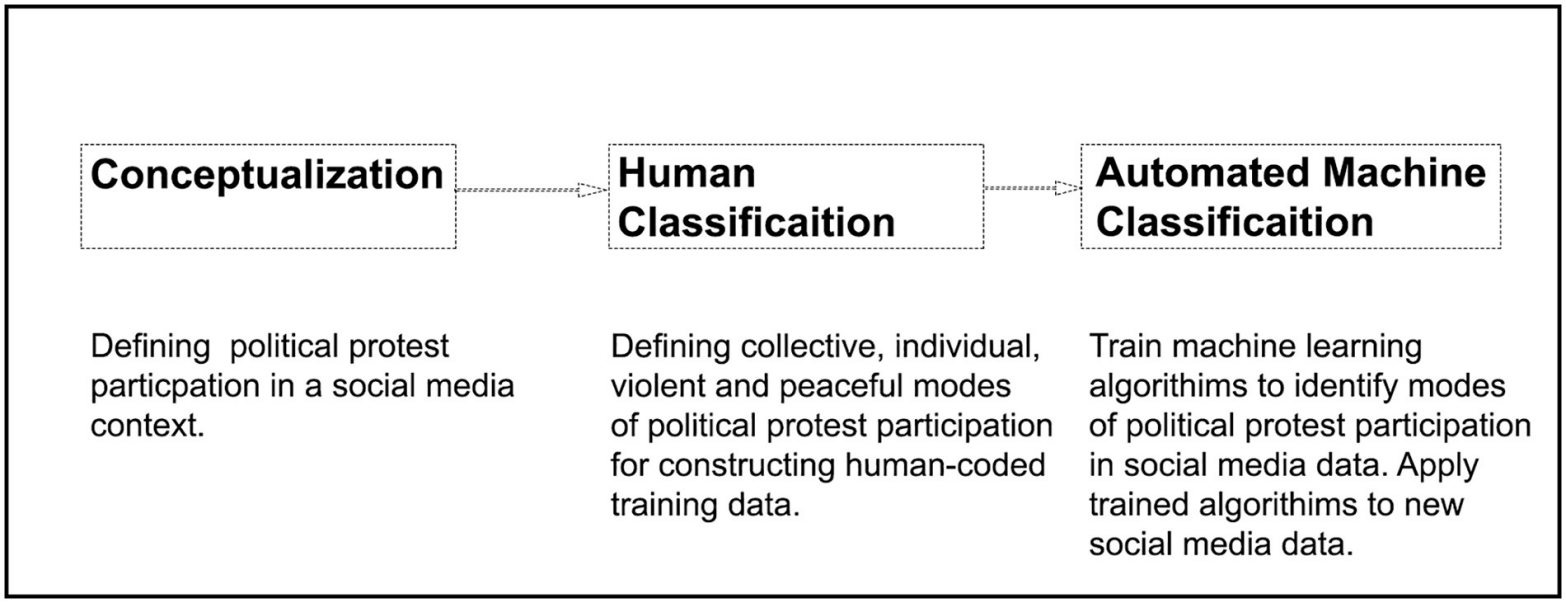
Overview of the process of identifying political protest participation from social media data. Each of these processes are described in more detail below.

As we discuss below, a political participation framework developed by Jan Van Deth in 2014 [10] and definitions of what constitutes violent collective action described by Tilly in his 2003 work *The Politics of Violent Collective Action* are instructive here. Finally, once this classification scheme is developed and a sufficient volume of social media data has been labeled by humans, machine learning algorithms can then automate the application of this classification scheme to new social media data. Fig 1 provides an overview of this process.

### Conceptualization

Conceptualization requires first identifying what constitutes political protest participation on social media more broadly. Once we have grasped this, we can use this information to understand how tweets, the social media data source used for this study, relate to the peaceful and violent forms of political participation that we are interested in. Recent work by Jan van Deth is instructive here. In a 2014 piece, van Deth develops an “operational definition of political participation” comprised of a clear set of decision rules which can be used to identify, and draw boundaries around, activity which constitutes “political participation”. These rules are summarized as answers to the following 7 questions:

1. Are we dealing with behavior?
2. Is the activity voluntary?
3. Is the activity done by citizens?
4. Is the activity located in the sphere of government/state/politics?
5. Is the activity targeted at the sphere of government/state/politics?
6. Is the activity aimed at solving collective or community problems?
7. Is the activity used to express political aims and intentions of participants?

These decision rules are then further abstracted into three major categories of political participation, described as *minimalist*, *targeted* and *motivational* definitions.

Minimalist definitions encompass the first 4 rules above. If all 4 are met, indicated by van Deth as {1^+^, 2^+^, 3^+^, 4^+^}, we can confidently classify an action as a mode of political participation. If, however, {1^+^, 2^+^, 3^+^} are met but 4 is not, in other words if the activity is not explicitly *within* the sphere of government or politics itself, then classification of an act as political participation requires then that the act is *targeted* at the sphere of politics, the government or the state more broadly, which gives rise to rule 5 and an additional category of political participation described as the *targeted* definition.

It is in this arena of *targeted* participation that traditional notions of protest come into play. Under the umbrella of targeted participation are included citizen demonstrations, blocking streets, painting slogans, flash mobs and other similar activities. As we describe below, representations of political protest participation as manifested through social media that we are concerned with focus primarily on affirmative answers to rules 1, 2, 3 and 5 to the exclusion of 4. Again borrowing van Deth’s terminology, *minimal* political protest participation on social media for our purposes entails phenomena which satisfy the minimal criteria for political protest participation as defined by the set of all actions manifested on social media which can be categorized within the set of actions {1^+^, 2^+^, 3^+^, 4^−^, 5^+^}. We provide some examples of these actions below.

In situations in which rules {1^+^, 2^+^, 3^+^} are satisfied but {4^−^, 5^−^} are not, in other words that the activity is neither located in nor targeted directly toward politics or the state, van Deth argues that an act can still be classified as political participation if it satisfies rule 6 and is aimed at solving collective or community problems. Examples of such activities include participation in acts which are aimed at community improvement (crime reduction, local schools, etc) through activities such as volunteering, bake sales and the like. The final rule, which encompasses the *motivational* definition of political participation includes acts which may not have traditionally been considered political participation because of their individualized nature but can be classified as political participation since the acts are used to express the political aims of the participants.

Under rule {1^+^, 2^+^, 3^+^, 4^−^, 5^−^, 6^+^} are included acts such as “buycotts” or “boycotts” where the purchase, failure to purchase or even destruction of consumer products is used to express a political aim. A recent example of a type of phenomenon falling into the *motivated definition* category includes reactions to a contract between the *Nike* footwear company and the politically controversial San Francisco 49ers quarterback Colin Kapernick who famously refused to stand during the U.S. Pledge of Allegiance traditionally recited before American football games. These actions by *Nike* led to the public destruction of *Nike* products through tweets and Facebook posts and vows never to purchase *Nike* products again by certain individuals. Our classification scheme, as we described below, is not concerned with such activities.

### Human classification of social media data: Framework

As we mention above we are concerned with identifying political protest participation in the context of social media data. Within van Deth conceptual framework, then, we are concerned with identifying tweets which represent the {1^+^, 2^+^, 3^+^, 4^−^, 5^+^} *targeted definition* of van Deth’s rules. Here, we develop a system which allows us to identify tweets relevant to the targeted definition to build a *training data set* that is subsequently coded by humans provided with instructions to make classification decisions using these rules. This training dataset is finally used to train a series of machine learning algorithms which allows us to extend this human coding framework through automatically labelling tweets related to both peaceful and violent forms of political protest activity. Because of its ability to make predictions using a broad variety of data types and to handle massive quantities of data, machine learning can be fruitfully employed as an automated sorting and tagging tool for tweets related to protest activity [11]. Below we describe how this framework was conceptualized, how it was used to build the training dataset and finally how this training dataset was used to train the algorithms which forms the basis of the software described above.

Building a classification scheme for identifying political protest participation on social media requires first understanding how the act of contributing content on social media, such as tweeting in our case, relates to political protest participation. We begin this analysis by considering what participation on social media entails more broadly. On social media, individuals post content (text, images, etc.) with the explicit knowledge and intention that others will be engaging with the information in some way. Looking back at van Deth’s first three rules for political participation which comprise the minimalist definition (i.e. “(1) Are we dealing with behavior?; (2) Is the activity voluntary; (3) Is the activity done by citizens.”), it is clear that tweeting, in general, satisfies these rules.

Starting with rule 1, that we are dealing with behavior, tweeting clearly is behavior which requires a conscious effort on the part of the tweeter when that tweeter is a human. Whether tweeting is a behavior when it is done by bots is a question that we do not discuss here, but note that it can also be argued that since bots require programming, tweets which are produced by bots can also be considered “behavior” in the traditional sense that is indirectly tied to the tweeter. To tweet, or post anything on social media requires that a conscious effort be made to compose a post/tweet, include relevant references (i.e. hashtags, individuals referenced etc.) and to submit the tweet for public scrutiny. Regarding rule 2, that the activity be voluntary, outside of exceptional circumstances in which individuals are paid to tweet on behalf of organizations, tweeting is generally understood as a voluntary activity engaged in by others for various ends [12].

Finally, regarding rule 3, that the activity is done by citizens, in the arena of social media it is not necessarily true for all, or even most tweets. Social media platforms such as Twitter, Facebook and Instagram are used by citizens for the purpose of communication, but also by governments, political and business organizations for a variety of purposes. Accordingly, the first goal of our human classification task is to ensure that we restrict the universe of tweets for our training data, to those that belong only to citizens and exclude those tweets produced by corporate or state entities.

To utilize these rules in the following framework we must first define A as the set of all possible activities on social media, and for any a∈A define the *standard minimalist forms* function *m*(*a*), which maps *a* to its set of rules. For example, supposing *a* represents a buycott activity, one would have *m*(*a*) = {1^+^, 2^+^, 3^+^, 4^−^, 5^−^, 6^+^}. With the function *m*, these three rules help us now arrive at our first *minimalist* definition for political participation on social media P:

**Definition 1**. The *minimalist definition for political participation on social media* requires that the social media activity (tweeting, posting etc), *a*, be in the subset, a∈P, of all activities on social media A, such that

$$\begin{array}{c} P=\{a\in A|\left\{1^{+},2^{+},3^{+}\right\}\cap m\left(a\right)=\left\{1^{+},2^{+},3^{+}\right\}\}. \end{array}$$

Thus, actions outside of P, which do not satisfy this minimalist definition for political participation fall outside of our study. Examples of excluded activities include tweeting by government organizations or politicians to promote policies, policy positions or for advertisement purposes in addition to tweets by corporate entities selling products.

As we mention above, the goal of our study is not to only define political participation on social media, but to specifically identify political *protest* participation as it manifests on social media. To accomplish this we turn to van Deth’s targeted definitions encapsulated in rules 4 and 5. Rule 4 asks whether “the activity is located in the sphere of government/state/politics” which includes activities such as “casting a vote, submitting an official petition or supporting a party or candidate.” While such activities can, in theory, take place on social media, our concern with identifying political protest participation precludes their inclusion.

Turning to rule 5, which we mentioned above is where the ordinary definition of political protest participation is located, the question asked is whether “the activity [is] targeted at the sphere of government/state/politics.” As with ordinary political protest participation the answer to this for our purposes is in the affirmative. Consequently, political protest participation within the sphere of social media is a subset of *targeted political participation* manifested T in a social media context described in more detail below.

**Definition 2**. In addition to meeting the minimalist criteria defined above political protest participation on social media requires that the social media activity (tweeting, posting etc) be in the subset of *targeted political participation*:

$$\begin{array}{c} T=\{a\in P|\left\{4^{-},5^{+}\right\}\cap m\left(a\right)=\left\{4^{-},5^{+}\right\}\}. \end{array}$$

**Fig 2:**
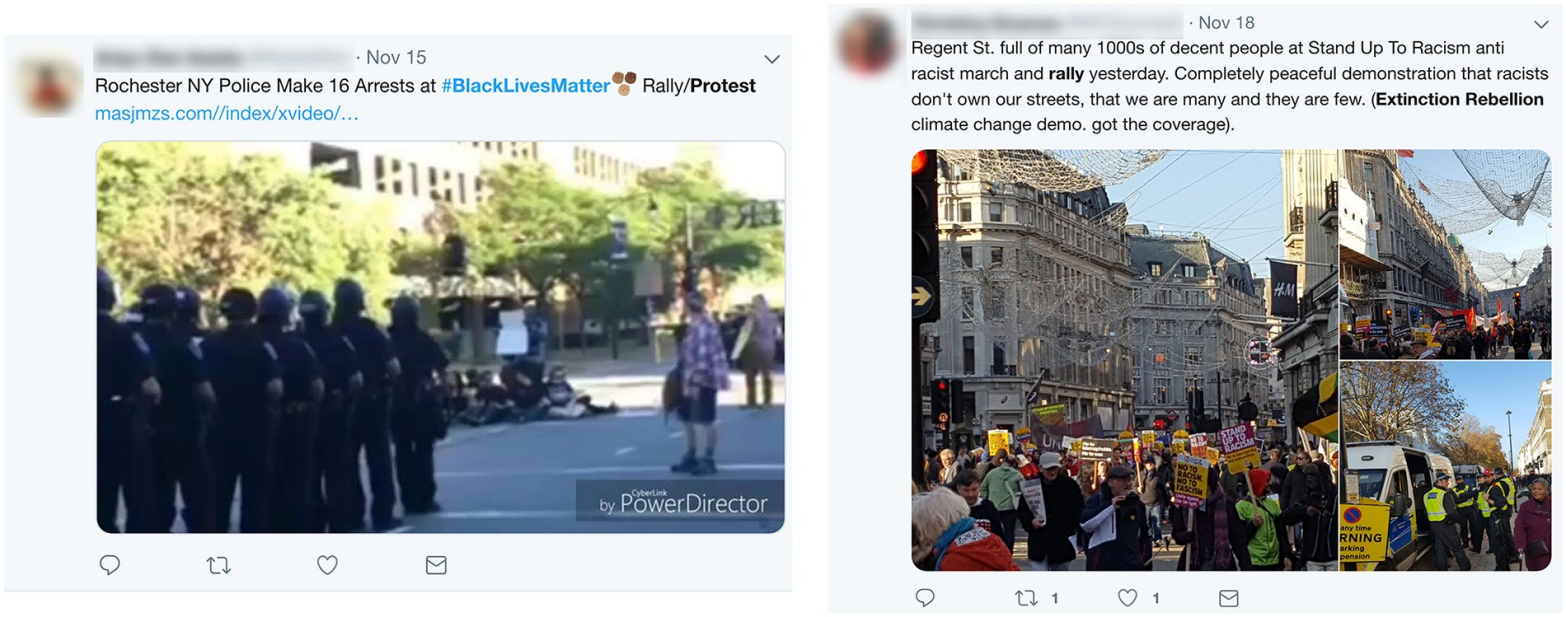
Examples of tweets illustrating *collective* forms of political protest participation.

In van Deth’s conceptual framework, this includes protest related activities such as demonstrations, blocking streets, painting slogans, flash mobs and so on. Here, we seek to identify manifestations of these *collective* political protest activities as expressed through tweets, a classification which we term *collective political protest actions* represented by the set of actions C. Examples of collective political protest actions as they would manifest on twitter include tweets such as those in Fig 2 which essentially serve as “reports” of *targeted political participation*.

To define refinements of T (like C) formally, we turn to Charles Tilly’s work *The Politics of Collective Violence*. Here, Tilly defines collective violence as actions which necessarily entail a different set of rules:

1. immediately inflict(s) physical damage on persons and/or objects (“damage” includes forcible seizure of persons of objects over restraint or resistance)
2. involves at least two perpetrators of damage; and
3. *results at least in part from coordination among persons who perform the damaging acts*. (Tilly 3)

**Fig 3:**
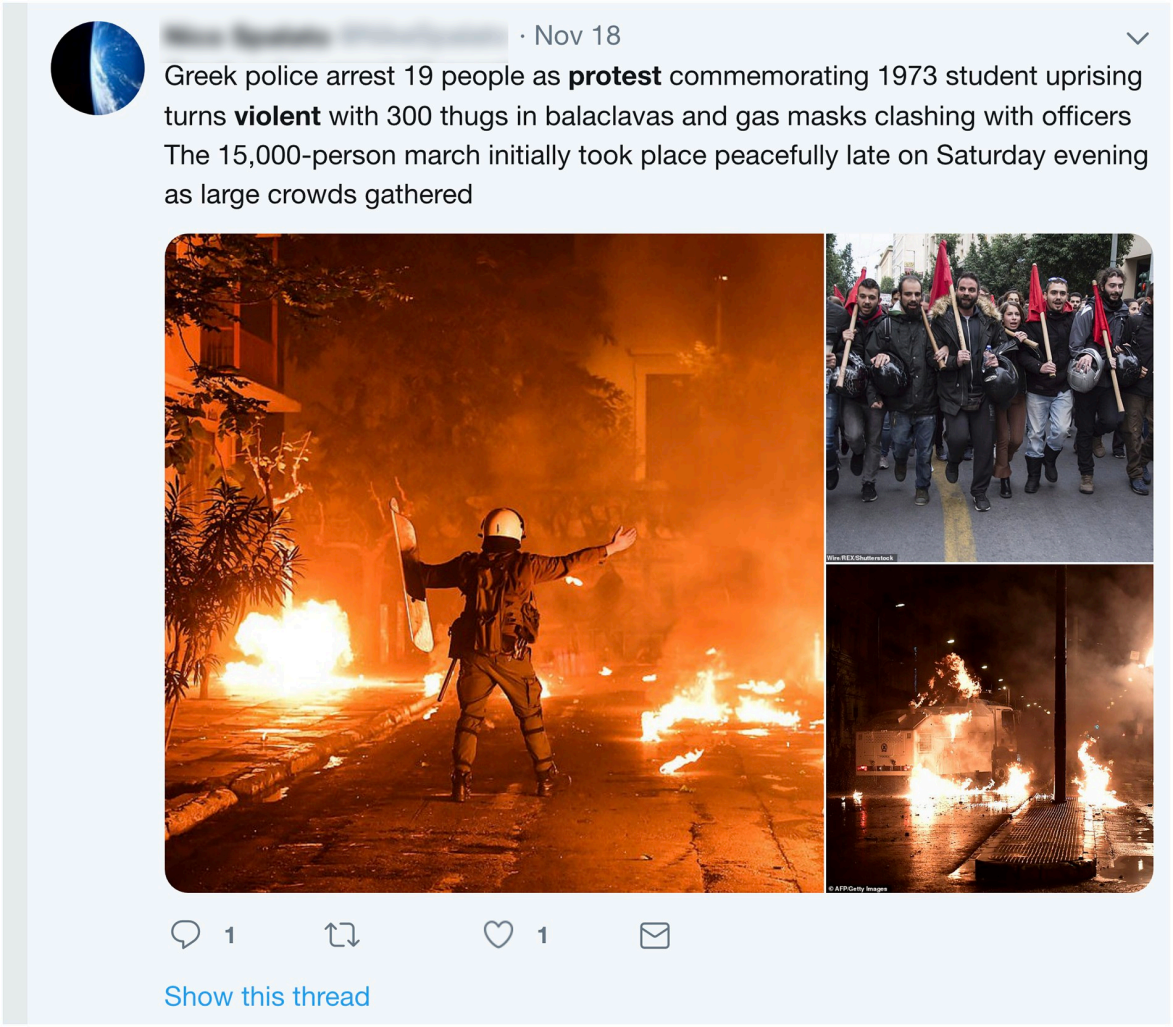
An example of a tweet defined in our framework as *collective force*.

The satisfaction of all three of Tilly’s rules therefore provides us with a refinement related to C, i.e., those actions which are collective *and* violent. For instance, the tweet in Fig 3 which documents coordinated property destruction within the context of political protest participation, falls squarely into this category. But, of what nature are the political activities in T that do not satisfy all three of *Tilly’s* rules? If Tilly’s first rule is not met, then the considered activity is still political in nature and collective, but does not entail violence, or perhaps more appropriately for our discussion, *force*, as some activities in Tilly’s definition are not ‘violent’, per se. Thus, we consider Tilly’s first rule as separating activities as being either *peaceful* or *forceful*, and define accordingly:

**Definition 3**. The set of targeted political participation activities on social media satisfying Tilly’s first rule (immediately inflicting physical damage or force to objects and/or persons) are defined to as *forceful* (F), and have compliment in T consisting of all *peaceful* activities: H=T\F.

**Fig 4:**
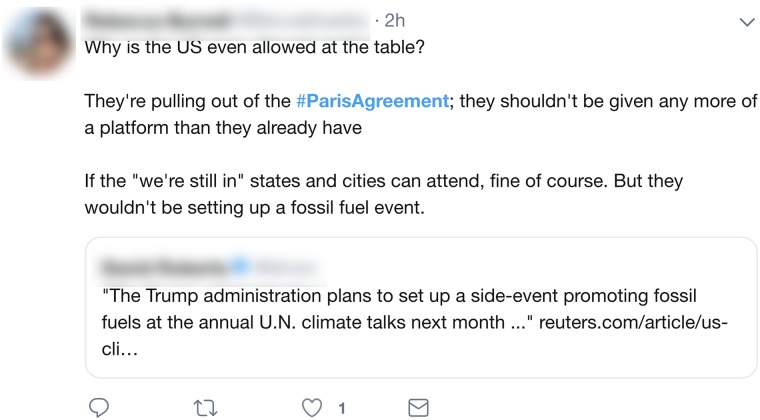
Example of a tweet illustrating *individual* or *singular* political protest participation on Twitter, expressing support for the Paris Agreement on climate change.

While collective political protest actions comprise a large swath of political protest participation on social media, these platforms also enable individual forms of political protest participation which we are interested in capturing. Specifically, they allow users to engage in and express political participation targeted at government/politics/state at the *individual* as well as the collective level. For example, individuals may tweet to bring awareness to or solutions for pressing political issues directed at government as in Fig 4. These individual political protest actions are another form of targeted political participation which we are interested in identifying through tweets. So, just as Tilly’s first rule might not be met, one can consider examples in which one or both of the second or third are not. For any of these cases, the ‘collective’ nature of Tilly’s definition is no longer satisfied, thus providing us with our distinction between collective C and individual I actions:

**Definition 4**. The set of targeted political participation activities on social media satisfying Tilly’s second *and* third rules (coordinated involvement of two or more persons) are defined to as *collective* (C), and have compliment in T consisting of all *individual* activities: I=T\C.

While Tilly’s rules allow for political protest participation on social media to be refined along two dimensions—agency and execution of power—these two dimensions can certainly interact in non-trivial ways. More precisely, while C∩I=F∩H=⌀ holds, activities may be found that fall under four combinations, namely C∩F (Tilly’s fully satisfied definition), C∩H, I∩F, and I∩H. However, considering our own terminology around the relaxation of Tilly’s ‘violence’ to include damages such as forcible seizure, we refer to these refinements as the collections of *collective force* (C∩F), *collective peace* (C∩H), *individual force* (I∩F), and *individual peace* (I∩H) activities in T.

**Fig 5:**
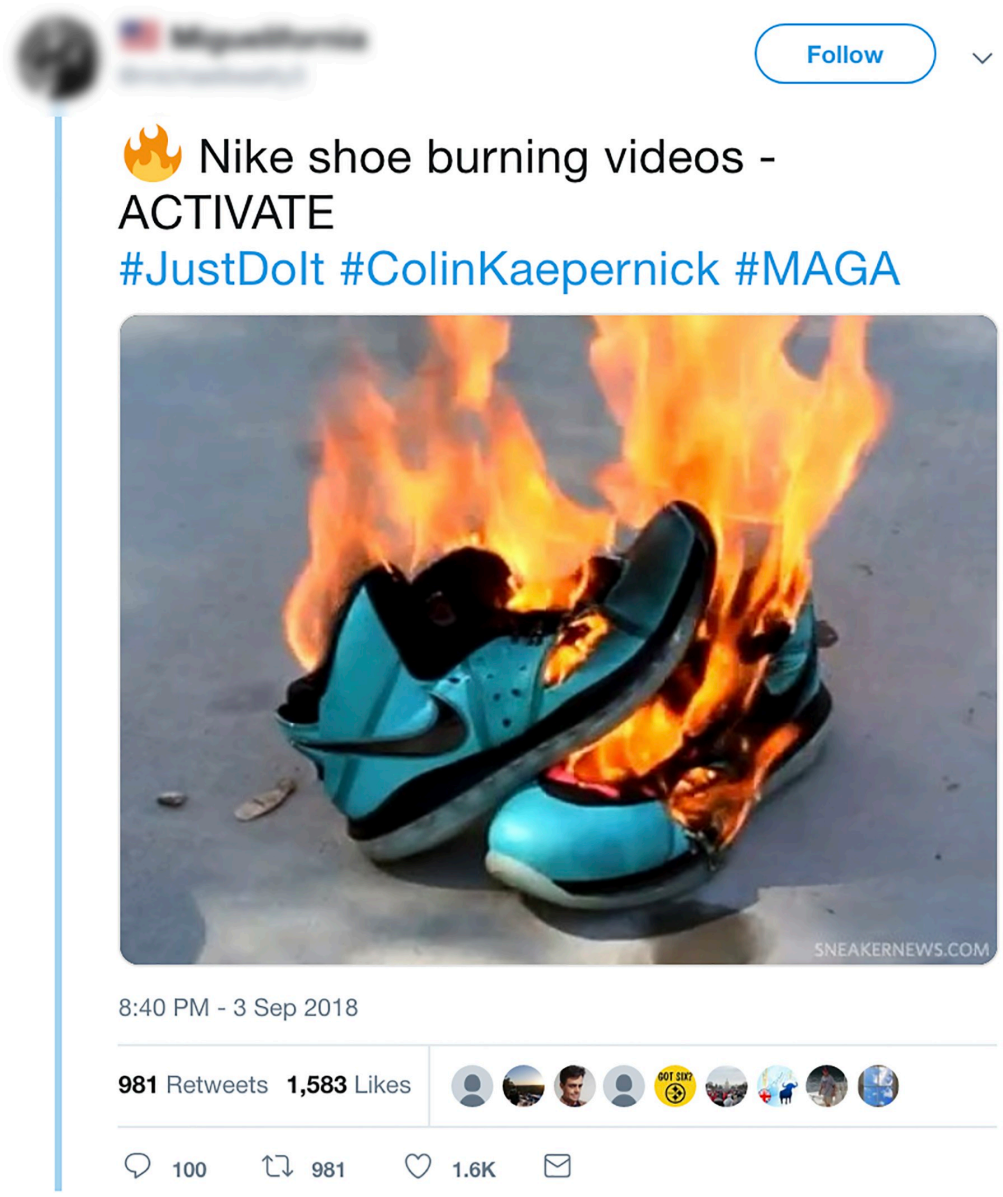
An example of a tweet defined in our framework as *singular force*.

Examples of *collective peace* include tweets such as those in Fig 2 which describe political protest participation that does not entail the infliction of physical damage to property or persons. In the social media context, the broader category of individual, or singular political protest participation includes acts in which individuals tweet about individual level activities targeted toward government/politics/the state. One example of such singular political protest participation includes tweets by an individual expressing concern with or support for a cause such as climate change as in Fig 4. Thus, under the above definitions the tweet in Fig 4 describes a tweet which would be categorized as *singular peace*. An example of a tweet characterizing singular force includes the tweet in Fig 5 in which an individual is setting a paid of *Nike* shoes on fire as an act of political protest after the company signed a contract with Colin Kapernick who kneeled during the national anthem at an American football game in an act of protest against racial inequality in the United States.

**Fig 6:**
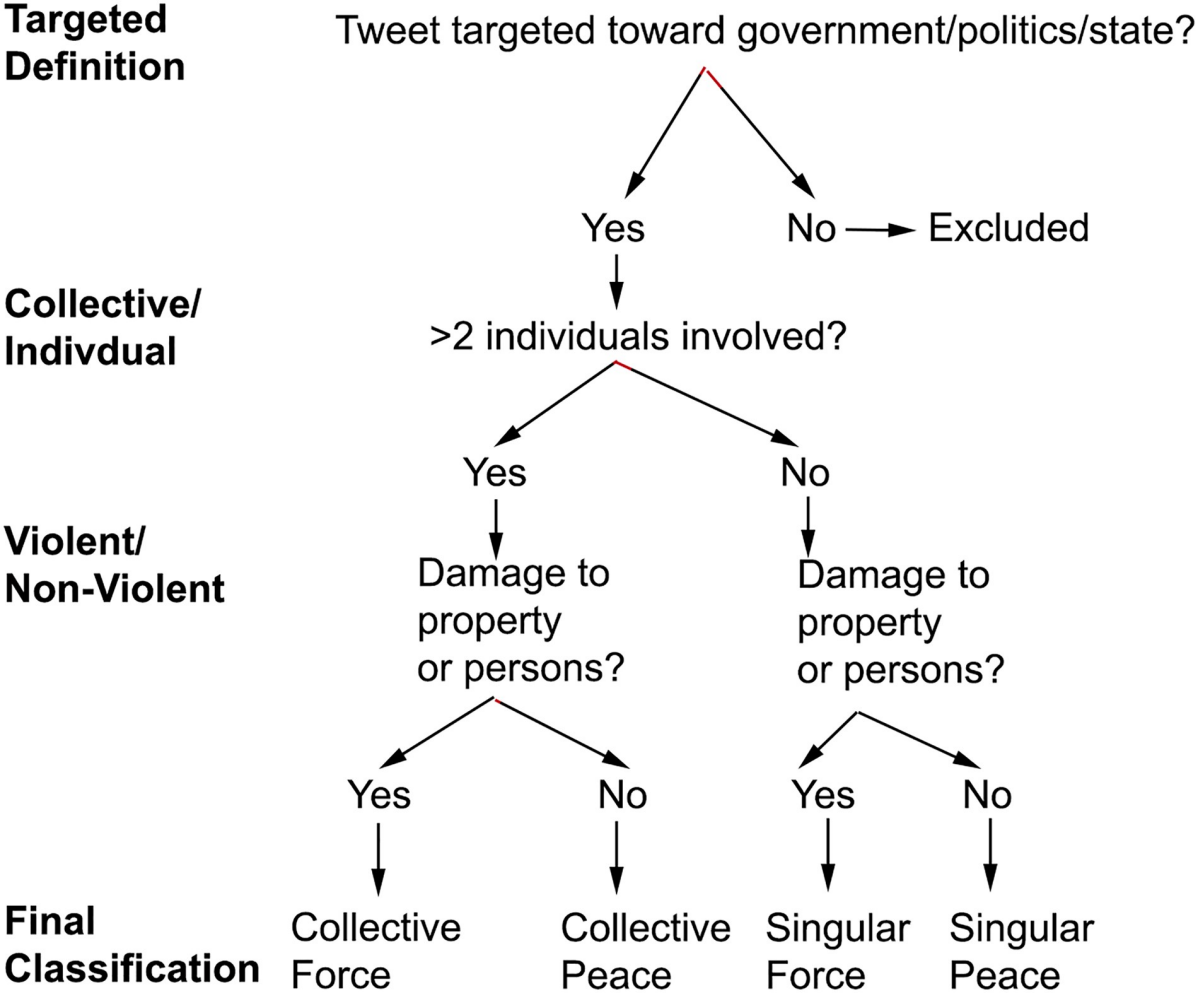
Summary of the logic of human classification decisions used to construct the training data.

Putting all of these definitions together, Fig 6 summarizes the series of decisions rules used by human coders to classify each of the tweets in our training data which we described in more detail in the subsequent section, into each of the four political protest participation categories. Using these guidelines and a sampling procedure which we describe in the following section, we constructed the training data which was used to built our automated tweet classification software.

## Human classification of social media data: Implementation

To explore the measurement of political protest participation under our classification framework derived above, we utilize a database of over 600 million geographically-tagged messages from the Twitter social network collected over the period April 1, 2014–April 30, 2015, which most notably covers the beginning of the Black Lives Matter (BLM) movement. This dataset was collected from Twitter’s public (spritzer) API.“API” is an acronym which stands for “Application Programming Interface.” An API is a set of communication protocols which allows other machines to communicate and extract information from a central server. As we describe in greater detail below, the subset of tweets relevant to political protest participation as defined above were first identified using *Associated Press (AP)* image metadata, specifically using the time and location of photographs identified by the AP being related to the coverage of protest activity. This was done to ensure that tweets will contain information about a highly diverse array of political protest participation around the globe.

**Fig 7:**
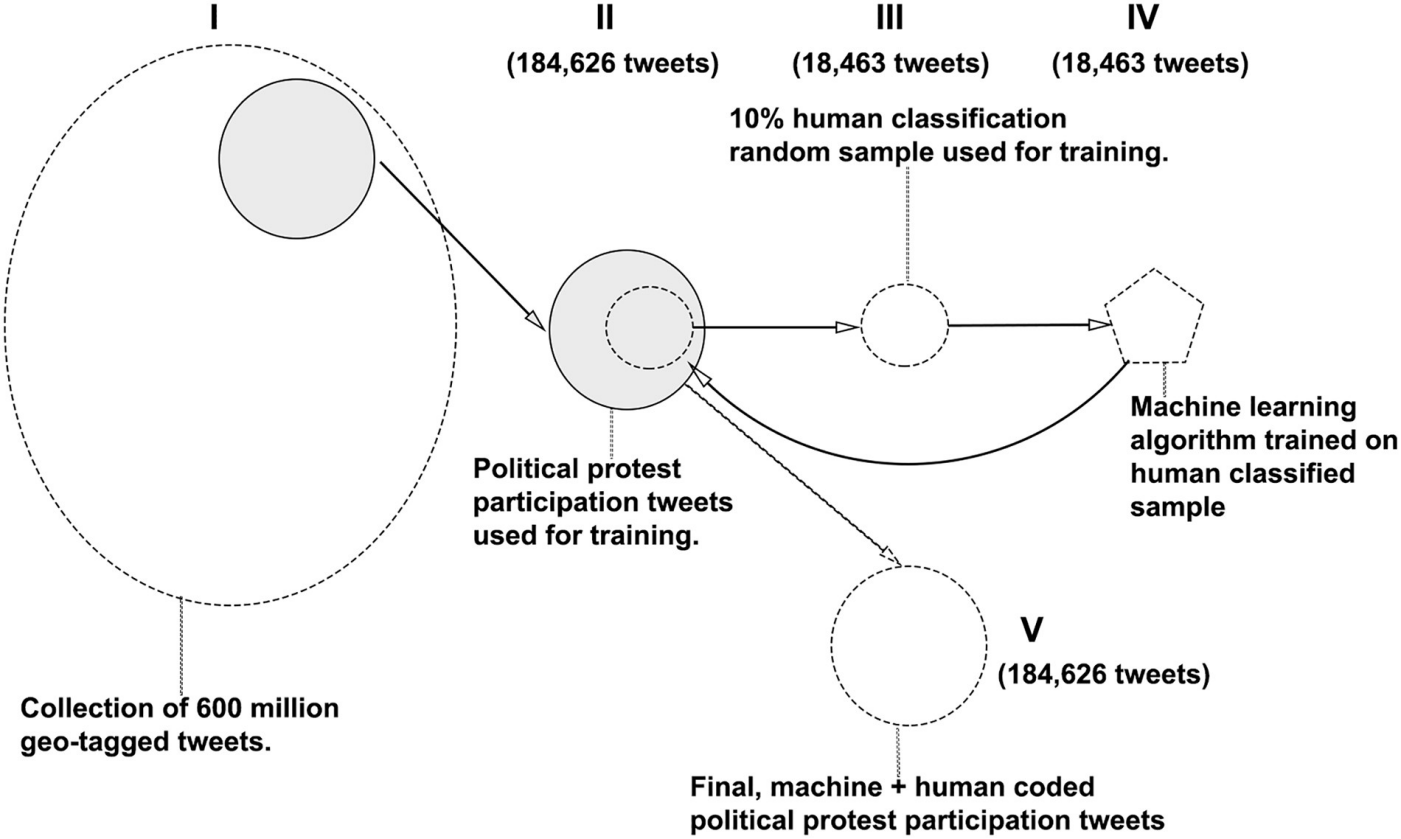
Overview of the process of classifying tweets into each of the 4 forms of political protest participation discussed above.

Fig 7 provides an overview of the process of transforming this large 600 million tweet database to database of classified political protest participation tweets based on our framework above. Stages **I**-**III** involve human classification and are described in this section, while stages **IV**-**V** involve automated machine classification and are described in the subsequent section.

At the time, when a user opted in for location tagging from a mobile device, a tweet sent would automatically be accompanied by high-precision latitude and longitude coordinates. Since then, Twitter enacted a policy that resulted in the adjustment of their system to default the option of soft-locations, which users specify (for example, one could set their location to Philadelphia and then go on to tweet from anywhere else in the world, with tweet meta-data always listed as Philadelphia). Since the rate at which tweets were geo-tagged was approximately 1% at the time of collection, and the (1% stream) public API was restricted to only location-tagged tweets, it is arguable that this dataset constitutes a near-complete collection of geo-tagged tweets during this period.

On account of the vastness of the Twitter database, we construct a filter that enables us to identify sets of Tweets more likely related to be related to protest activity over the time period by our original tweet database (Stage **II**). While this kind of information can, in theory, be accessed through newspaper accounts of protest activity, identifying the locations and exact times that protests took place around the world using newspaper accounts would require collecting a massive database of newspaper articles in different languages from around the world and is subject to the same sampling biases favoring violent political protest participation discussed above.

Instead, we leveraged an *Associated Press* image database containing thousands of images and extracted the relevant metadata contained therein, which included the exact time and location that photos of protest activity were taken (see Supplementary Materials for details). With this information, we build a tweet filter for our coding of political protest participation (assuming this sample to be particularly potent in representation of the four modes of political protest participation as defined above) [13]. This filtering process yielded a total of 184,626 tweets extracted from the original database, used for both training and out-of-sample validation purposes described below.

Using 10% (18,463) of the tweets sampled from the AP-filtered protest times and locations, a group of undergraduate human coders at the University of California, Berkeley under our supervision coded tweets individually for the presence of the four modes of political protest participation using the series of decision rules captured in Fig 6 above.

### Potential demographic biases of our data

Through the process selecting tweets for classification as represented through Stages **I** and **II**. A number of biases are introduced into our final software product which must be accounted for. Beginning with our original database of 600 million geo-tagged tweets, this database contains tweets from users whom either (1) actively wanted their location tracked or; (2) were unaware that their location was being tracked and did not turn location services off. While we do not have the demographic information from this sample of users, a study conducted by Pew Research in May, 2013 of social media users who have their geo-location services on is informative.

Among users of social media sampled, users who had geo-location services turned on were roughly the same in term of gender (30% of males and 30% of females) and educational attainment (31% of “High School or Less”, 31% of “Some College” and 29% of “College +”). Where users with geo-location services turned on differed however was in terms of age, household income and “urbanity”, the term used in the *Pew Research* study. Within these categories, users with geo-location services turned on tended to be younger. For social media users ages 18-29, 32% of users had geo-location services on, for ages 30-49, 34% had geo-location services on while for users ages 50-64 only 26% had geo-location services on while for users 65 years of age and older only 18% had geo-location services on. These users also tended to have either low or high-incomes ($30,000-$49,999: 26% vs. <$30,000: 32%, $50,000-$74,999: 33% and $75,000+: 36%.) and came primarily from urban or suburban locations rather than rural. Among rural users only 24% had geo-location services turned compared with 28% of urban and 35% of suburban social media users.

In sum, the database that we draw from and identify political protest activity on Twitter with is likely to contain users that are younger, who are either low or high income earners and who live in urban and suburban areas. Of these factors, perhaps the most concerning is the significantly smaller proportion of rural tweeters, suggesting the we are unlikely to detect political protest activity that happens to be located in more rural areas.

## Automated machine learning classification of social media data

Above, we described how we acquired and labeled the training data represented in Stages **I**–**III** in Fig 7 we now describe the core machine learning methodology that is at the heart of our automated tweet classification software which comprise Stages **VI** and **V** in Fig 7. We use the coded tweets collected and labeled as input for a series naïve Bayes classifiers (one for each political protest classification category as discussed above in the previous sections and in Fig 6, which, for each of the four modes of political protest participation, run in parallel. These standard naïve Bayes classifiers are also modified with an enhanced input feature space of both single and multiword expressions. This process is accomplished through a recently developed [14] multiword expression segmentation method which bases our classifiers on integrated collections of words and phrases. We refer to the resulting systems as “adept” Bayes classifiers, whose features have two notable advantages:

1. independent, semantic accuracy, and
2. improved out-of-context human interpretability.

Thus, for example, basing the adept classifier on the expression *tear gas* sidesteps confounding statistical effects in the frequencies of the words *tear* and *gas*, and at the same time may be interpreted by a diagnostician to appropriately mean a crowd suppression device. Interpreted separately, these words might indicate an epidemic of indigestion.

In addition to improving the Bayes classifier used in our experiments, the usage of phrases as features allows for greater interpretability of classifications. Our adept Bayes classifier has an advantage of being explorable, as a “white box” method that can be opened to show the features most relevant to classifications. In particular, looking at a document as a bag of phrases *d* = *w*_1_, *w*_2_, ⋯, *w*_*N*_, counted with frequencies *f*(*w*_1_), *f*(*w*_2_), ⋯, *f*(*w*_*N*_), their impact on the adept Bayes classification is largely due to the likelihood function, Λ (determined in training), which, often computed as a sum of logarithms, is linear in frequencies:

$$\begin{array}{c} -\sum_{i=1}^{N}f\left(w_{i}\right){\text{log}}_{10}\Lambda \left(w_{i}|c\right). \end{array}$$ (1)

Note: *c* is the mode’s presence (positive/negative), and the terms are negated for an entropic framing. If a diagnostician wishes to understand why a tweet was classified as positive (*c*_+_) over negative (*c*_−_), the difference may be computed:

$$\begin{array}{c} -\sum_{i=1}^{N}f\left(w_{i}\right)({\text{log}}_{10}\Lambda \left(w_{i}|c_{+}\right)-{\text{log}}_{10}\Lambda \left(w_{i}|c_{-}\right)). \end{array}$$ (2)

Such a difference affords a ranking of features (tweet terms) by the (absolute) terms of the sum, i.e., each word, *w*_*i*_: *i* = 1, ⋯, *N*, can be compared according to the relative impact on classification:

$$\begin{array}{c} f\left(w_{i}\right)({\text{log}}_{10}\Lambda \left(w_{i}|c_{+}\right)-{\text{log}}_{10}\Lambda \left(w_{i}|c_{-}\right)). \end{array}$$ (3)

**Fig 8:**
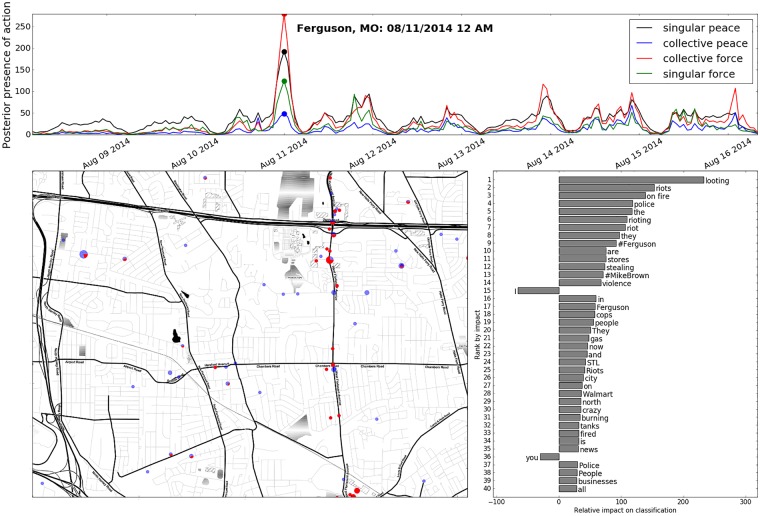
Above. Time series showing the total presence of social action types in Ferguson, MO the week after the shooting of Michael Brown by Officer Darren Wilson on August 9, 2014. The presence of each action type is determined by our adept Bayes classifier and measured as the sum of posterior probabilities over all tweets from each hour in the plotted span of time. Left. Map of Ferguson, MO depicting clusters of collective force activity over one hour around 12 AM, on August 11th. The size of each cluster-circle represents the area from which tweets emerged (not the number of tweets contained), and the portion of each circle colored red indicates the portion of tweets classified to represent the collective force action. Right. A phrase shift showing the most impactful features present in all tweets classified as being representative of collective force. Phrases on the right pull the classifier toward a positive classification, and phrases on the left pull the classifier towards a negative classification.

**Fig 9:**
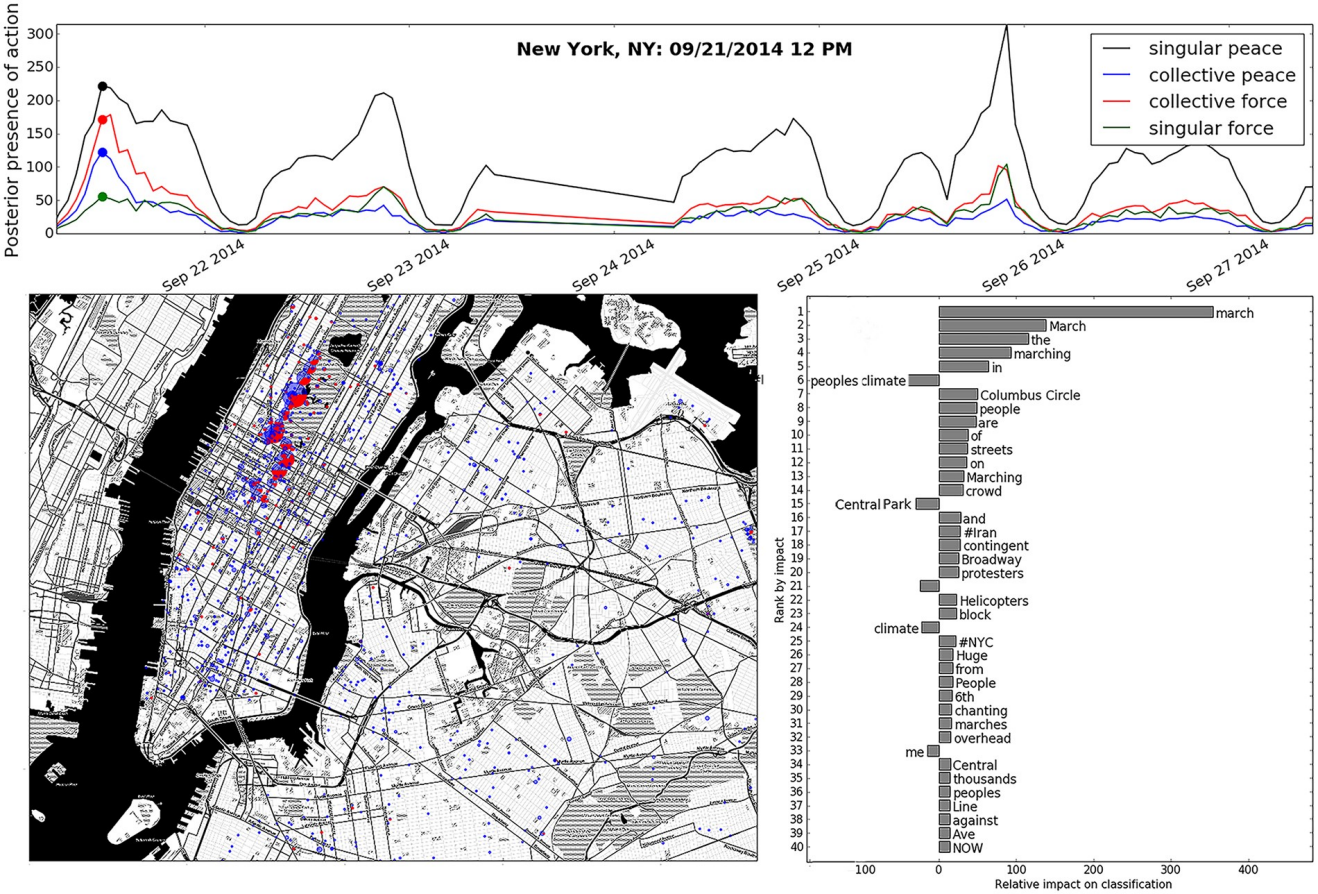
Above. A time series showing the total presence of social action types during the week of the People’s Climate March which began on September 21, 2014. Left. Map of New York, NY depicting clusters of collective force and collective peace activity over one hour around 12 PM on September 21st during a climate change protest. The size of each cluster-circle represents the area from which tweets emerged (not the number of tweets contained), and the portion of each circle colored red indicates the portion of tweets classified to represent the collective force action. Right. A phrase shift showing the most impactful features present in all tweets classified as being representative of collective force. Phrases on the right pull the classifier toward a positive classification, and phrases on the left pull the classifier towards a negative classification.

**Fig 10:**
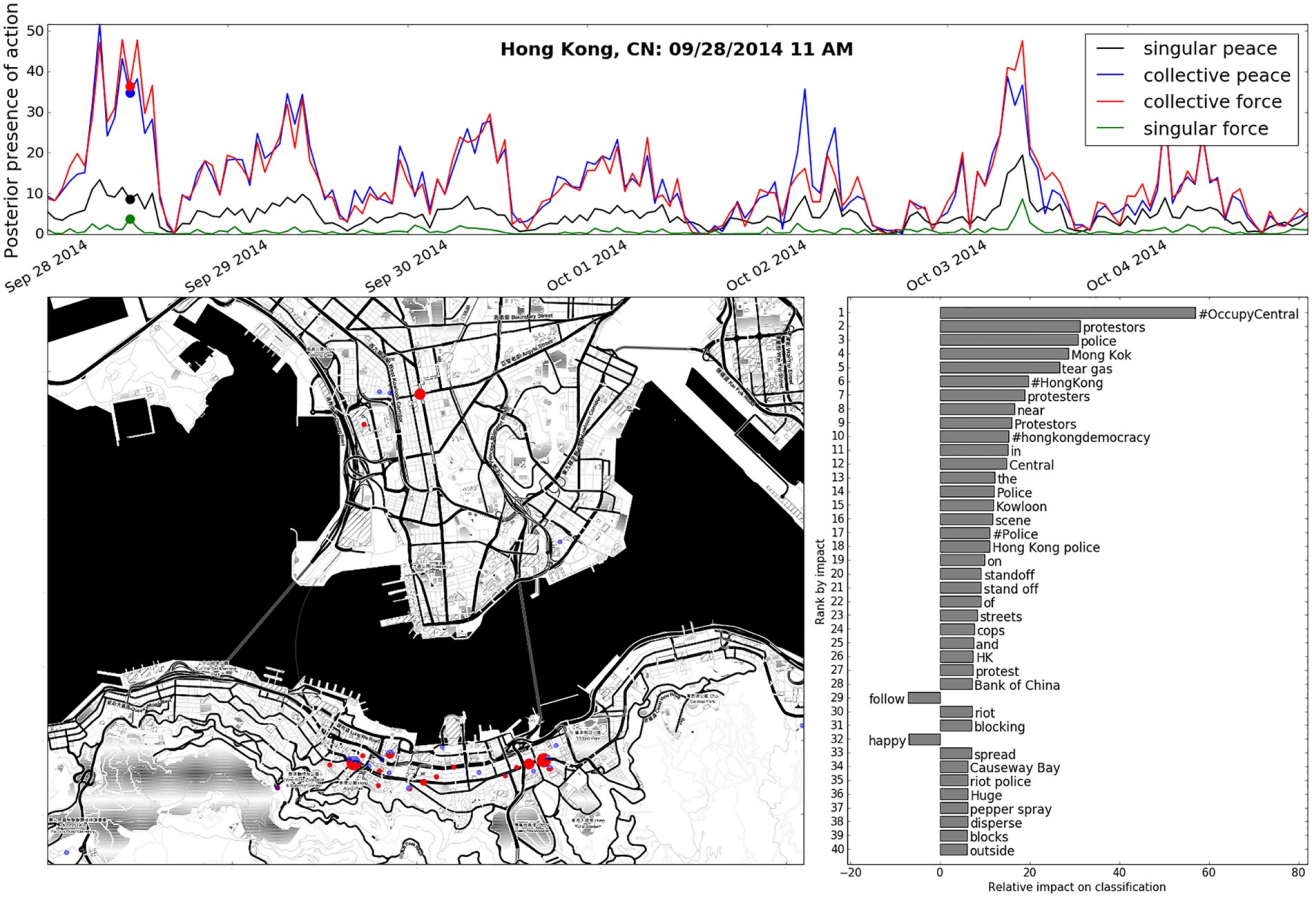
Above. A time series showing the total presence of social action types during the week of the Occupy Central with Love and Peace movement which began on September 28th, 2014 Left. Map of Hong Kong depicting clusters of collective force activity over one hour around 11 AM, on September 29th. Right. A phrase shift showing the most impactful features present in all tweets classified as being representative of collective force. Note: points and bars represent analogous quantities to those in Figs 8 and 9.

Thus, for diagnostic value we display the ranked values of Eq 3 along a vertical bar plot, which we call a phrase shift (see Figs 8, 9 and 10).

After training our adept Bayes classifier on coded data from Stage **IV** we examine its performance by performing a tenfold cross-validation on the coded tweets data set. The results of this validation are recorded in Table 1. Treating the Bayes posterior probability as a tunable threshold for classification, we measure precision and recall, and optimize the threshold probability over *F*_1_ to tune each given classifier. Observing these results, we see that, with the exception of *singular force*, each of the modes of political protest action are well predicted with a *F*_1_ statistic of above 0.50. While classifier performance at predicting collective peace and singular force is lower, we do see that the most prevalent type of action, singular peace, is predicted well. When the classifiers are collapsed to less-specific types of action (Collective, Singular, Peace, and Force) performance decreases from the best cases (singular peace and collective force), but when all action types are combined (All), we see a significant performance improvement in all measures.

**Table 1:** Tenfold cross-validation results from application of the naïve Bayes classifier for the different modes of political protest action. Each labeled row indicates the number of positive (i.e. identified as one of the four modes of political protest participation) in the training data set (**Abundance**), and the *F*_1_-optimal posterior probability (**Threshold**) for classification, in addition to its the corresponding values of precision (**P**), recall (**R**), and combined *F*_1_. *Precision* refers to the probability that the trained classifier will identify a *true positive* result when applied to new data. *Recall* refers to the probability that the classifier will identify a positive result out of a sample of politively classified tweets. The *F*_1_ statistic combines *precision* and *recall* to provide an overall measure of classifier quality. Out-of-domain evaluations are presented parenthetically, adjacent to their corresponding in-domain values.

Action	Abundance	Threshold	P	R	*F*_1_
**Collective force**	795 (99)	0.08 (0.35)	74.08 (64.44)	76.01 (58.59)	74.94 (61.38)
**Collective peace**	474 (111)	0.78 (0.04)	51.92 (45.87)	55.25 (45.05)	53.29 (45.45)
**Singular force**	351 (6)	0.92 (0.42)	57.39 (0.25)	41.19 (16.67)	47.38 (20)
**Singular peace**	1,823 (96)	0.85 (0.11)	73.52 (44.68)	67.61 (43.75)	70.38 (44.21)
**Collective**	1,116 (168)	0.79 (0.17)	74.54 (75.91)	68.8 (61.90)	71.48 (68.20)
**Singular**	1,951 (101)	0.87 (0.36)	71.44 (41.18)	68.57 (55.45)	69.90 (47.26)
**Force**	1,107 (103)	0.71 (0.21)	66.94 (60.19)	67.54 (63.11)	67.22 (61.61)
**Peace**	2,092 (178)	0.88 (0.23)	71.5 (53.69)	72.2 (61.24)	71.78 (57.22)
**All**	2,596 (226)	0.93 (0.24)	80.71 (65.52)	74.42 (75.66)	77.39 (70.23)

While we cannot quantify our model’s performance in application to real-time, streaming data yet, we can hint at its performance on out-of-domain data by separating known distinct events in the training data. In addition to the BLM movement, the coded data significantly cover a portion of the Hong Kong democracy protests. Using the data from Hong Kong for testing, and all other data for training, we see somewhat different results (see Table 1, parentheticals), and generalize to note some potential challenges with using this data (that only covers a limited set of events) to build a classifier and apply it to data representing unknown and unforeseen events.

First, the subject matter (democracy) from the Hong Kong tweets is very different from that of the BLM movement (institutionalized racism), making the discourse present in the singular peace test tweets largely unrelated to that from training. As a result, this previously predictable category now exhibits substantially decreased performance. Furthermore, of the nearly 900 tweets coded from Hong Kong, only 6 were found to be representative of singular force, so there is essentially nothing to predict for this category.

For the collective actions we see very different numbers, especially for collective force. This is likely as a result of the similar collective tactics employed on both sides of both movements (e.g., blockades, non-lethal pacification, etc.). When the different action type are collapsed, we see more and more performance improvements, indicating that the collapsed categories may be the most reliable. However, since the Hong Kong tweets are actually part of the training of the overall classifier, we note that the performance of our model when applied to real-time data will likely be better than that reported in Table 1 (parentheticals).

## Interpretation

To demonstrate how our classifier can be used by researchers and the public to learn about violent and peaceful forms of political protest action, we apply our trained classifier to data from outside of training, taken from Ferguson, MO during the initial wave of protest activity, over August of 2014. In Fig 8 we plot a time series of this period, showing the abundance of the four types of social action, as measured by the sum of posterior probabilities of all tweets under the application of our classifiers. Here, it can be seen that the largest spikes occurred on the first night of protesting (Aug. 10^th^). While singular peace (black line) exhibits a substantial, periodic signature even under normal circumstances (the discourse it covers is regular and common), collective force (red line) emerges aberrantly during the protest events, overshadowing the presence of the other action types.

Taking a closer look at the presence of collective force at the largest spike, we zoom in to a map of the first night of protesting in Fig 8, and plot clusters of tweets with the positive classifications represented as proportional areas. Here, we can see a larger cluster just south of the freeway, on West Florissant Avenue, which corresponds to the time and location of the burning of the QuikTrip convenience store and gas station (set to fire by protestors). This action is actually hinted at in the phrase shift (bar plot, right), by terms such as “burning” and “on fire.” While the first night of protesting was violent and unexpected, the actions that took place were spread out, and involved fewer mass confrontations with the police, which later became more militarized and can be observed in figures depicting subsequent evenings.

We additionally present the result of our model’s application to the Hong Kong democracy protests that lasted for approximately two months in the fall of 2014. On the map in Fig 10, we see clusters of collective force activity at the three main protest sites: Admiralty, Causeway Bay, and Mong Kok. Tactics similar to those reported by Twitter users during the Ferguson protests were employed by the Hong Kong police as well, as is indicated by a phrase shift (Fig 10, right) that shows highly-impactive phrases such as “tear gas,” “stand off,” and “riot police.” So for the collective force category, we see a large degree of accord in the lexical features that indicate the presence of the action (which we quantify below, in Table 1 (parentheticals)), indicating the possibility of applicability to out-of-domain data, and future events.

## Building a protest activity database

While the literature discusses many aspects of protests, one of the central political questions surrounding protest activity involves its effects on public opinion [15] and its ability to influence policymakers. The effects of protest activity on public opinion are especially important to understand because the former typically influences the latter. To construct measures of protest activity researchers have mostly relied on compiling databases of newspaper coverage of protest activity. While these databases arguably capture some of the most impactful protest activity, they have limited the study of protests to those picked up by media sources.

**Fig 11:**
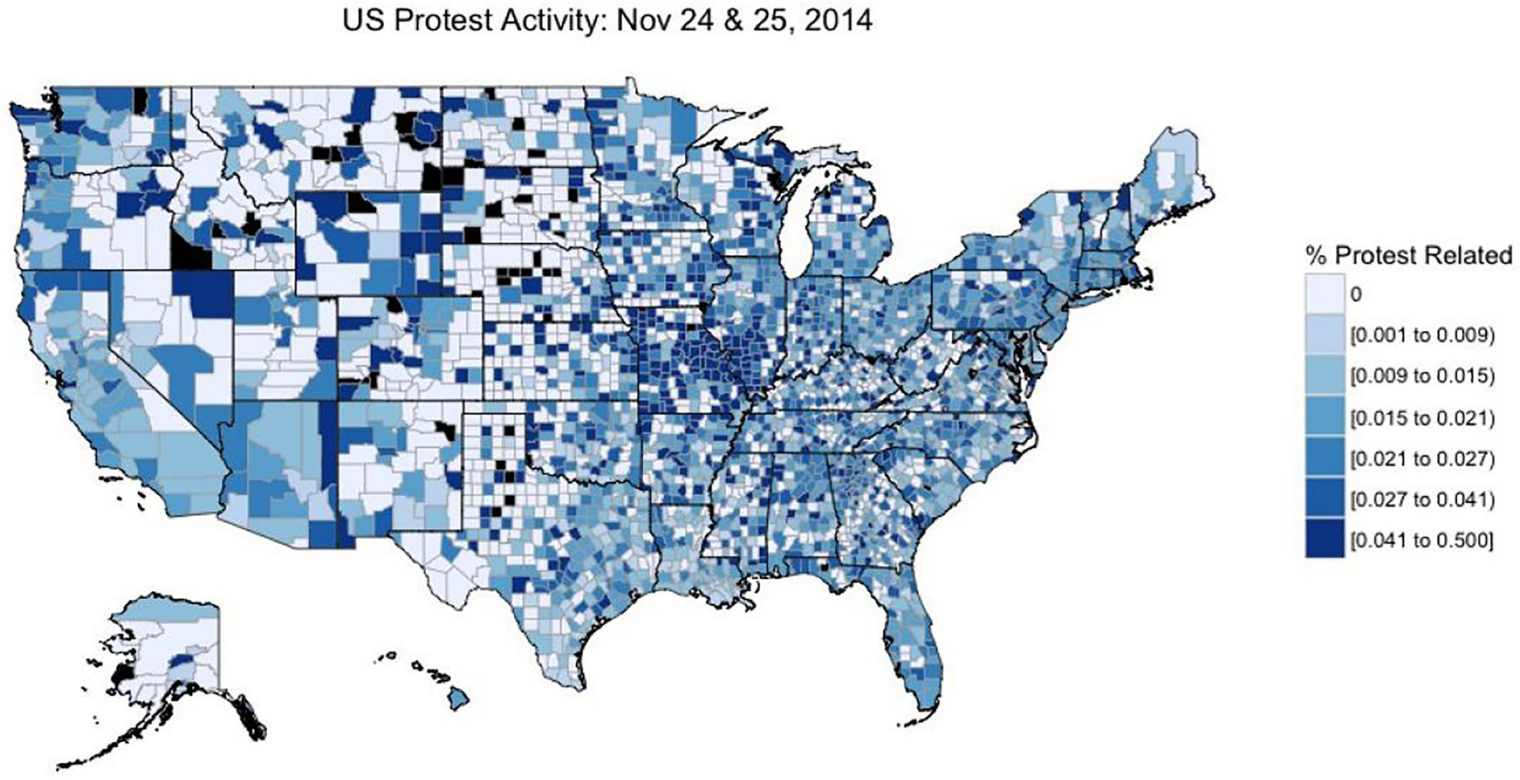
Ferguson-related political action across US counties on November 24th and 25th, 2014 as measured by the percentage of Tweets related to any form of political action.

Here, we demonstrate how our software can be used by researchers to build a database of measurable protest activity at the county level in the United States. Using our software, we explore patterns of protest activity across the United States shortly after the grand jury acquittal of Officer Darren Wilson in Ferguson, MO on November 24th and construct measures of protest activity using a subsample of 3.5 million classified Tweets. The Fig 11 map contains measures of overall political activity across the United States—notably centralized in Missouri—after the acquittal of Darren Wilson on November 24th, 2014.

## Discussion

As political protest participation in the digital age becomes increasingly represented on social media, a theory which is able to map textual data and metadata onto events occurring on the ground provides a means by which these data can be harnessed to better understand the evolution of modern political protest participation in its peaceful and violent manifestations. In this paper, we build upon conceptual frameworks identified by van Deth and Tilly for identifying peaceful and violent forms of political protest participation which we argue accomplishes this task and demonstrate how this framework can be used to identify the forms of political protest participation for social media data in a machine learning context. While the software and model discussed above were constructed using Twitter data, this method can be applied to any text-based form of real-time streaming social media (i.e. *Facebook* posts, *Instagram* metadata etc.). As such, this paper adds to a growing body of work focused on the automatic detection of events from social media streams. The information we have depicted in Figs 8, 9, and 10 serve as examples of the diagnostic utility that our developed framework and methods can provide.

### Copyright and compliance

In collecting this data and producing this document, we have complied with all of the data collection terms and conditions stipulated by the *Associated Press (AP) Images Database* hosted by the University of California, Berkeley Library System and with all of the data collection terms and conditions stipulated as stipulated by *Twitter Inc.*.

All map images provided in the document were produced by the *Mapbox* package in *Python*: https://github.com/mapbox/mapbox-sdk-py. Tiles produced by the *Mapbox* package can be used under the Creative Commons 3.0 license (CC BY 3.0). More about this can be found here: http://maps.stamen.com.

Any questions or concerns regarding these matters should be directed to:

L. Jason Anastasopoulos

Email: ljanastas@uga.edu

## Supporting information

S1 File Supporting information and Python code for “A scalable machine learning approach for measuring violent and peaceful forms of political protest participation with social media data”. (PDF)
