# Supplementary material for: A scalable machine learning approach for measuring violent and peaceful forms of political protest participation with social media data
Source: PLoS One. 2019 Mar 19;14(3):e0212834. doi: 10.1371/journal.pone.0212834 (PMC6424401; doi:10.1371/journal.pone.0212834)
Supplement: S1 File — (PDF) [file pone.0212834.s001.pdf]

Supporting information for "A scalable machine learning approach for measuring violent and peaceful forms of political protest participation with social media data"

1 Materials and Methods

1.1 Identifying Protest Time and Location with Associated Press (AP) Image Metadata

The adept Bayes classifier built as part of this study was trained on human-coded Tweets labeled with each of the four types of social actions: Singular Peace, Collective Peace, Singular Force and Collective Force. Because the database of 600 million geo-coded Tweets which we had available to us was simply too large for human coders to sift through for the purposes of identifying each of the social actions discussed above, it was necessary for us to narrow down sets of Tweets that would have the highest probability of corresponding to each of the four social actions. While those studying social actions in the past have used searches through media accounts of protest activity there are a number of issues related to using this method which our method of utilizing AP image metadata solve.

First, as discussed above written media accounts of protest activity tend to be biased towards very large scale protests and social actions that often involve violence. Since newspaper databases are mostly in English as well, coverage of protest activity will be biased towards protests occurring in the English-speaking world. AP photographers, on the other hand, cover a much wider variety of protest activity both within English speaking countries and outside of English speaking countries.

This is because many AP photographers work on a freelance basis and will take photographs of events that may or may not be subsequently written about by larger corporate news sources such as CNN, Fox, NBC, CBS and others [1]. Thus, AP images and image metadata related to protest activity will include a much wider breadth and depth of protest and social action activity than will traditional news sources. This, in turn, provides us with a much wider scope for our training data and ultimately a much more useful and accurate classifier.

Second, while newspaper accounts require exhaustive searches through texts to determine the time and place that a social action or protest occurred, Associated Press image metadata contains information about exactly when and where a photo of a particular protest or social action was snapped. Furthermore, all photographs were visually verified as including some type of social action.

As mentioned above we used AP image metadata to identify clusters of Tweets that were more likely to be related to the each of the social actions defined in the paper. To do this we first queried the full AP Image Database which we had subscription access to through the U.C. Berkeley Library by searching for every photo containing the tag "protest" between April 1st, 2014 and April 30th, 2015, the date range that we had geocoded Tweets available for. This search yielded a total of 5,156 photos which were subsequently downloaded by ourselves and 5 U.C. Berkeley undergraduate assistants and compiled in an image database. While we are able to release metadata extracted from these images, Associated Press copyright restrictions prevent us from releasing the database containing the images themselves.

For each image, metadata which included a caption, photographer, date and time

that the photo was taken, country, city and state (where applicable) was available in the metadata.

The Python code which was used to extract and format the image metadata is shown below. Please note that all files in the code below refer to machine readable *.json* versions of the *.csv* data files which we included as part of this submission for purposes of exposition.

```
import json, re, os, iptcinfo
from iptcinfo import c_datasets, c_datasets_r, IPTCInfo

root = "~/Dropbox/URAP_APImages/"
outfile = root+"data/events.json"

people = [
  "student_1", "student_2", "student_3",\
  "student_4", "student_5", "student_6", "student_7"]

events = {}
missing = {}
for person in people:
    imagenames = os.listdir(root+person+"/images/")
    imagenames = [x for x in imagenames if 'jpg' in x]
    f = open(root+person+"/images/img_info.json",'w')
    for image in imagenames:
        try:
            iptc = dict(IPTCInfo(image).data)
            data = {}
            for numkey in iptc:
                data[c_datasets[numkey]] = iptc[numkey]
             f.writelines(json.dumps(data) + '\n')
        except:
            missing.setdefault(person,[])
            missing[person].append(image)
    f.close()

    lineNum = 0
    try:
        f = open(root+person+"/images/img_info.json", "r")
        lineNum = 0
        for line in f:
            lineNum += 1
            try:
                data = json.loads(line)
                city = data["city"]
                caption = data["caption/abstract"]
                date = data["date created"]
                events.setdefault(city, {})
                events[city].setdefault("boxes", [])
                events[city].setdefault("captions", {})
                events[city]["captions"].setdefault(date, [])
                if caption not in events[city]["captions"][date]:
                    events[city]["captions"][date].append(caption)
            except:
                pass

        f.close()
    except:
        print person+": no info file!"
os.chdir("~/projects/protest-project/")
f = open("events.json", "w")
f.writelines(json.dumps(events))
f.close()
f = open("missing_images.json","w")
f.writelines(json.dumps(missing))
```

Fig A Sample of tweets and social action codes in JSON format used for the social action coding task.

```
f.close()
```

Code A Python code for AP Image Metadata Extraction

After Code A was run, the collected image metadata held within **events.json** was edited by hand to associate the "city" field to appropriate geographic units within the geo-coded Tweets database, which is annotated with codes for the GADM global administrative boundaries. See http://www.gadm.org/version2 for more information. The contents of the resulting GADM-associated database of image metadata are dumped into CSV format under the file **APprotestevents-metadata.csv** for convenience.

1.2 Extracting samples of geo-coded tweets Using AP metadata

Using the GADM-associated AP image metadata database **events.json** discussed above, the geo-coded Twitter database was queried and tweets were extracted using geographic and time parameter ranges which corresponded to event data listed in **APprotestevents-metadata.csv**. While alignment of the extracted data to the geo-coded Twitter database was straightforward for temporal information (times were simply converted from GMT to EST), association for geographic data was somewhat more complicated. Generally, the AP images were tagged with city-level spatial information, while the geo-coded Twitter database was tagged for US county-level equivalents. This resulted in each city being coded by hand for overlapping counties, which resulted in different levels of refinement for different cities. For example, while San Francisco was mapped to its name-identical county, New York City was identified with the five boroughs, and each of Oakland and Berkeley were associated with their superset, Alameda county. Ultimately, the effects of this imperfect mapping were most significantly seen at the time of coding the filtered Twitter date for social actions. In particular, for the small cities that are subsets of large, populous counties, like Ferguson MO., the filtering resulted in samples that were more "watered down" in terms of social action (since more unrelated tweets were included from the surrounding areas).

After extraction, tweets were reformatted into a series of JSON formatted files which were then distributed to each of the 5 U.C. Berkeley undergraduate students who were subsequently instructed about how to code each of the tweets that they were assigned according to the four social action categories that we described in the paper. Following this, the student-coded tweets were comprehensively audited for consistency and then merged to form the final coded data set.

A sample of the completed Tweet database as viewed by the coders is presented in Fig A. The Python code which was used to query the Tweet database and format relevant tweets for human coding is below.

```
import datetime, os

f = open("events.json", "w")
f.writelines(json.dumps(events))
f.close()

for city in events:
    dts = []
    runs = []
    for d in events[city]:
        if d != "boxes" and d != "runs":
```

```
            for t in events[city][d]:
                ttime = d[0:4]+"-"+d[4:6]+"-"+d[6:8]+"-"+t[0:2]+"-"+t[2:4]
                dts.append(ttime)
    dts = sorted(dts)
    pt = datetime.datetime.strptime(dts[0],'%Y-%m-%d-%H-%M')
    i = 0
    runs.append(["NA","NA"])
    runs[i][0] = (pt - datetime.timedelta(hours=5.25)).strftime('%Y-%m-%d-%H-%M')
    for dt in dts:
        dt = datetime.datetime.strptime(dt,'%Y-%m-%d-%H-%M')
        if dt - pt > datetime.timedelta(hours=0.5):
            runs[i][1] = (pt - datetime.timedelta(hours=4.75)).strftime('%Y-%m-%d-%H-%M')
            i += 1
            runs.append(["NA","NA"])
            runs[i][0] = (dt - datetime.timedelta(hours=5.25)).strftime('%Y-%m-%d-%H-%M')
        pt = dt
    if runs[i][1] == "NA":
        runs[i][1] = (pt - datetime.timedelta(hours=4.75)).strftime('%Y-%m-%d-%H-%M')
    events[city]["runs"] = runs

    if len(events[city]["boxes"]):
        boxes = events[city]["boxes"]
        outfile = "~/projects/protest-project/data/tweets/"+city+".txt"
        f = open(outfile,"w")
        f.close()
        for run in runs:
            start = run[0]
            end = run[1]
            cd = "cd ~/tools/perl/pullTweets; "
            cmd = "perl bin/pullTweets.pl -tblucm start="+start+" end="+end+" boxes=["+",".join(boxes)+"] languages=[en] >> \""+outfile+"\""
            os.system(cd+cmd)
```

Code B Python code using AP image metadata to query geo-coded Tweet database.

The Python code which was used to prepare the tweets for human coding is shown below.

```
import re, json
import random as ra

with open("~/projects/protest-project/events.json","r") as f:
    events = json.loads(f.read())

data = {}
raw = {}
for city in events:
    infile = "~/projects/protest-project/data/tweets/"+city+".txt"
    try:
        f = open(infile,"r")
        i = 0
        for line in f:
            line = line.strip()
            if re.match("^(.*?)\t(.*?)\t(.*?)\t(.*?)\t(.*?)\t(.*?)$",line):
                i += 1
                time, box, coords, lang, user, tweet = re.split("\t", line)
                ID = city + "-" + str(i).zfill(5)
                ID = re.sub(" ","_",ID)
                raw[ID] = {
                    "time": time,
```

```
                    "box": box,
                    "coords": coords,
                    "user": user,
                    "tweet": tweet
                }
                data[ID] = {
                    "tweet": tweet,
                    "codes": {
                        "singular-peace": 0,
                        "collective-peace": 0,
                        "collective-force": 0,
                        "singular-force": 0
                    }
                }
        f.close()
    except:
        print "no data on "+city+"!"
f = open("~/projects/protest-project/data/surveys/allTweets_pm15m.json","w")
f.writelines(json.dumps(raw))
f.close

ra.seed(0)
tweeKeys = list(data.keys())
ra.shuffle(tweeKeys)
i = 0
j = 0
batch = {}
for ID in tweeKeys:
    i += 1
    batch[ID] = data[ID]
    if not i % 3000:
        j += 1
        f = open("~/projects/protest-project/data/surveys/batches/batch-"+str(j).zfill(3)+".json","w")
        f.writelines(json.dumps(batch))
        f.close()
        f = open("~/projects/protest-project/data/surveys/batches/batch-"+str(j).zfill(3)+".tsv","w")
        for ID in batch:
            line = ID+"\t[0,0,0,0]\t"+json.dumps(batch[ID]["tweet"])+"\n"
            f.write(line)
        f.close()

        batch = {}
        i = 0
if len(batch.keys()):
    j += 1
    f = open("~/projects/protest-project/data/surveys/batches/batch-"+str(j).zfill(3)+".json","w")
    f.writelines(json.dumps(batch))
    f.close()
    f = open("~/projects/protest-project/data/surveys/batches/batch-"+str(j).zfill(3)+".tsv","w")
    for ID in batch:
        line = ID+"\t[0,0,0,0]\t"+json.dumps(batch[ID]["tweet"])+"\n"
        f.writelines(line)
    f.close()
```

Code C Python code used to prepare tweets for human coding.

1.3 Building the labeled data database for adept Bayes classifier

After coding of the relevant tweets, tweets were compiled into a labeled tweet database containing 22,626 tweets which was used to train the adept Bayes classifier and assess classifier performance. The labeled tweet data used to train and test the classifier is included as part of the supplementary materials in this submission under the filename **Coded-Tweets-training.csv**.

1.4 Building and training the adept Bayes classifier

In addition to the substantive contribution of this paper, we provide a methodological innovation which improves upon a well known machine learning classifier known as naïve Bayes. While the naïve Bayes classifier is known to provide superior performance results and scalability in the context of text classification, a major drawback of the algorithm is the conditional independence assumption which assumes that words within documents are independent of each other. Although the naïve Bayes classifier performs very well for text analysis tasks despite the conditional independence assumption, many have theorized that relaxing this assumption can potentially improve performance even further [2].

Here, we relax the conditional independence assumption by incorporating a NLP phrase chunking method [3] that allows us to better meet the independence assumptions of the naïve Bayes classifier, resulting in an enhanced naïve Bayes classifier which we refer to as “adept” Bayes.

Code for implementing the adept Bayes classifier is shown below.

```
import json, re, sys, os
import numpy as np
import math as ma
import random as ra
from partitioner.methods import MWE
pa = MWE()

def countPhrases(message):
    counts = {"*": 0.}
    for MWE in pa.partition(message):
        phrase = "".join([block[1] for block in MWE])
        if re.search("[a-zA-Z]", phrase):
            counts.setdefault(phrase,0.)
            counts[phrase] += 1.0
            counts["*"] += 1.0
    return counts

def loadPhrases(record):
    codes = record["codes"]
    counts = countPhrases(record["tweet"])
    for code in codes:
        codeCounts = counts
        if codes[code]:
            codeCounts["_"+code] = 1.
            yield code,codeCounts
        else:
            codeCounts["_not."+code] = 1.
            yield "not."+code,codeCounts

def aggregateByPhrase(d1,d2):
    for key in d2:
        d1.setdefault(key,0)
        d1[key] += d2[key]
    return d1

```

```python
def findN(record):
    ID,counts = record
    yield "_ALLCODES",counts
    counts["_N"] = float(len(counts.keys()) - 1)
    yield ID,counts

def computeEntropies(record):
    code, entropies = record
    n = float(entropies["_N"])
    entropies["*"] = sum([entropies[phrase] for phrase in entropies\
                          if phrase != "_N" and phrase != "*" and phrase
                              != "_"+code])
    m = float(entropies["*"])
    del entropies["_N"]
    del entropies["*"]
    alpha = n/N

    for phrase in entropies:
        if not re.match("^_", phrase):
            entropy = -np.log2((entropies[phrase]+alpha)/(alpha*N+m))
            entropies[phrase] = entropy

    entropies["_DEFAULT"] = -np.log2(alpha/(alpha*N + m))
    return code,entropies

def processTweet(tweet):
    result = {
        "posteriors": {code: -prior_entropy[code] for code in
            prior_entropy},
        "M_words": 0.,
        "counts": {},
        "N": 0.,
        "M": 0.,
        "tweet": tweet
    }

    review_M = 0
    tempCounts = countPhrases(tweet)
    for phrase in tempCounts:
        if phrase != "*":
        result['counts'].setdefault(phrase,0)
        result['counts'][phrase] += tempCounts[phrase]

    todel = []
    for phrase in result['counts']:
        if phrase != "*":
            INLIKE = False
            for code in likelihoodEntropy:
                if likelihoodEntropy[code].get(phrase, False):
                    result['posteriors'][code] -= result['counts'][phrase
                        ]*likelihoodEntropy[code][phrase]
                    INLIKE = True
                if INLIKE:
                    result['M'] += result['counts'][phrase]
                    result['M_words'] += result['counts'][phrase]*len(re.
                        split(" ",phrase))
                    result['N'] += 1
                    for code in likelihoodEntropy:
                        if not likelihoodEntropy[code].get(phrase, False):
                            result['posteriors'][code] -= result['counts'
                                ][phrase]*likelihoodEntropy[code]["
                                _DEFAULT"]
                    result['counts'][phrase] = int(result['counts'][phrase
                        ])
                else:
                    todel.append(phrase)
```

```
    for phrase in todel:
        del result['counts'][phrase]

    newPosteriors = {}
    for code in result['posteriors']:
        code = re.split("\.",code)
        if len(code) == 2:
            code = code[1]
            newPosteriors.setdefault(code,{})
            newPosteriors[code]["0"] = result['posteriors'][code]
        else:
            code = code
            newPosteriors.setdefault(code,{})
            newPosteriors[code]["1"] = result['posteriors'][code]
    result['posteriors'] = newPosteriors

    for code in result['posteriors']:
        if len(result['posteriors'][code].keys()) == 2:
            P0 = 1./(1. + (2 ** (result['posteriors'][code]["1"] - result['posteriors'][code]["0"])))
            P1 = 1. - P0
            result['posteriors'][code] = {"0": P0, "1": P1}
        else:
            result['posteriors'][code] = {"0": 1., "1": 0.}

    result["M"] = int(result['M'])
    result["M_words"] = int(result['M_words'])

    return result
```

Code D Functional components of the adept Bayes classifier. A complete pipeline uses all but the last function, *processTweet*(), for training, and the final function for classification, as will be seen in the subsequent cross-validation code.

Using the coded data from Twitter, the model was trained as a parallel series of binary classifiers. In other words, a separate adept Bayes classifier was trained for each of the four types of social action (e.g., collective force, singular peace, etc.), in addition to the collapsed categories (i.e., collective, singular, force, peace, or any). This means that the processing of the coded data resulted in the training of 9 binary classifiers that can separately assess the presence of each type of social action, e.g., application of the classifier would predict that a tweet either is a representation of collective force, or is not a representation of collective force, while simultaneously predicting if the same tweet is a representation of singular peace, or is not a representation of singular peace, et cetera.

1.5 Assessing classifier performance

After training the adept Bayes classifier, we assesses precision, recall and F_1 statistics using tenfold cross-validation on the trained model.

The code used to produce the trained model and the classifier statistics are shown below.

```
codes = [
    "collective-force",
    "collective-peace",
    "singular-force",
    "singular-peace",
    "collective",
    "singular",
    "peace",
    "force",
    "action"
]
```

```
thresholds = [(x)/100. for x in range(1,100)]

records = []

with open("trainingData.json", "r") as f:
    for line in f:
        record = json.loads(line)
        oldcodes = dict(record["codes"])
        for code in oldcodes:
            for newcode in re.split("-", code):
                if not record["codes"].get(newcode, 0):
                    record["codes"][newcode] = oldcodes[code]
            if not record["codes"].get("action", 0):
                record["codes"]["action"] = oldcodes[code]
        records.append(record)

ra.seed(0)

ra.shuffle(records)

numfolds = 10
folds = []

foldsize = int((len(records)/numfolds) + 0.5)
if foldsize*numfolds < len(records):
    foldsize += 1
allresults = []
for foldnum in range(numfolds):
    trainingrecords = []
    testingrecords = []

    for k in range(numfolds):
        if k == foldnum:
            testingrecords.extend(list(records[k*foldsize:(k+1)*foldsize]))
        else:
            trainingrecords.extend(list(records[k*foldsize:(k+1)*foldsize]))

    ###### training
    ########################
    allcounts = {}
    for code in codes:
        allcounts[code] = {}
        allcounts["not."+code] = {}

    total_tweets = 0.
    for record in trainingrecords:
        total_tweets += 1.
        for k, v in loadPhrases(record):
            allcounts[k] = aggregateByPhrase(allcounts[k],v)

    for code in codes:
        allcounts["_ALLCODES"] = aggregateByPhrase(allcounts[code], allcounts["not."+code])
        break
    for ky in allcounts.keys():
        allcounts[ky]["_N"] = float(len(allcounts[ky].keys()) - 1)

    N = float(len(allcounts["_ALLCODES"].keys())) - 2.

    del allcounts["_ALLCODES"]

    entropies = []
    for ky in allcounts.keys():
```

```
        entropies.append(computeEntropies((ky, allcounts[ky])))

    likelihoodEntropy = {}
    prior_entropy = {}

    for x in entropies:
        code = x[0]
        entdict = x[1]
        priorNum = entdict["_"+code]
        prior_entropy[code] = priorNum
        del entdict["_"+code]
        likelihoodEntropy[code] = entdict

    for code in prior_entropy:
        prior_entropy[code] = -np.log2(prior_entropy[code]/total_tweets)
    ###### end training
    ########################

    ###### testing
    ########################
    totals = {}
    for code in codes:
        totals[code] = {}
        for thresh in thresholds:
            totals[code][str(thresh)] = {
                "TP": 0.,
                "FP": 0.,
                "FN": 0.,
                "TN": 0.
            }
    for record in testingrecords:
        result = processTweet(record["tweet"])
        for code in codes:
            truth = record["codes"][code]
            for thresh in thresholds:
                st = str(thresh)
                if result["posteriors"][code]["1"] >= thresh:
                    prediction = 1
                else:
                    prediction = 0

                if truth:
                    if prediction:
                        totals[code][st]["TP"] += 1.
                    else:
                        totals[code][st]["FN"] += 1.
                else:
                    if prediction:
                        totals[code][st]["FP"] += 1.
                    else:
                        totals[code][st]["TN"] += 1.

    for code in codes:
        for thresh in thresholds:
            st = str(thresh)
            try:
                totals[code][st]["TPR"] = totals[code][st]["TP"]/(totals[code][st]["TP"] + totals[code][st]["FN"])
            except:
                totals[code][st]["TPR"] = 0.
            try:
                totals[code][st]["PPV"] = totals[code][st]["TP"]/(totals[code][st]["TP"] + totals[code][st]["FP"])
            except:
                totals[code][st]["PPV"] = 0.
            try:
```

```
                totals[code][st]["F1"] = (2.*totals[code][st]["TPR"]*totals[code][st]["PPV"])/\
                                          (totals[code][st]["TPR"] + totals[code][st]["PPV"])
            except:
                totals[code][st]["F1"] = 0
            try:
                totals[code][st]["ACC"] = (totals[code][st]["TP"]+totals[code][st]["TN"])/\
                                           (totals[code][st]["TP"]+totals[code][st]["TN"]+\
                                            totals[code][st]["FP"]+totals[code][st]["FN"])
            except:
                totals[code][st]["ACC"] = 0

    print "the results for fold: "+str(foldnum)
    for code in codes:
        st = max([(totals[code][str(thresh)]["F1"], str(thresh)) for thresh in thresholds], key=lambda x: x[0])[1]
        totals[code]["thresh"] = st
        print code, st, totals[code][st]["PPV"], totals[code][st]["TPR"], totals[code][st]["F1"], totals[code][st]["ACC"]
    print
    allresults.append(totals)
    ########################

####### report the F1-optimal posterior threshold for each social action type
bestThresh = {}
for code in codes:
    bestThresh[code] = [0, 0]
    for thresh in thresholds:
        F1 = np.mean([totals[code][str(thresh)]["F1"] for totals in allresults])
        if F1 > bestThresh[code][1]:
            bestThresh[code] = [thresh,F1]
    thresh = bestThresh[code][0]
    print code, thresh, np.mean([totals[code][str(thresh)]["PPV"] for totals in allresults]),\
                        np.mean([totals[code][str(thresh)]["TPR"] for totals in allresults]),\
                        np.mean([totals[code][str(thresh)]["F1"] for totals in allresults])
```

Code E Python code used to produce tenfold cross-validation statistics for the adept Bayes classifier.

In addition to this, we sought to assess out-of-domain performance of the classifier for each type of action. To accomplish this, we trained the adept Bayes classifier on all coded data, except for those that were drawn from Hong Kong, which were used for testing. Under this setup, the classifier had no in-domain knowledge of the Hong Kong Democracy protests that were tested upon.

The Python code for the execution of the out-of-domain test is shown below.

```
codes = [
    "singular-peace",
    "collective-peace",
    "collective-force",
    "singular-force",
    "collective",
    "singular",
    "peace",
    "force",
    "action"
```

```
]

thresholds = [(x)/100. for x in range(1,100)]

trainingrecords = []
testingrecords = []

with open("trainingData.json", "r") as f:
    for line in f:
        record = json.loads(line)
        oldcodes = dict(record["codes"])
        for code in oldcodes:
            for newcode in re.split("-", code):
                if not record["codes"].get(newcode, 0):
                    record["codes"][newcode] = oldcodes[code]
            if not record["codes"].get("action", 0):
                record["codes"]["action"] = oldcodes[code]
        if re.match("Hong_Kong",record["ID"]):
            testingrecords.append(record)
        else:
            trainingrecords.append(record)

###### training
########################
allcounts = {}
for code in codes:
    allcounts[code] = {}
    allcounts["not."+code] = {}

total_tweets = 0.
for record in trainingrecords:
    total_tweets += 1.
    for k, v in loadPhrases(record):
        allcounts[k] = aggregateByPhrase(allcounts[k],v)

for code in codes:
    allcounts["_ALLCODES"] = aggregateByPhrase(allcounts[code], allcounts[
        "not."+code])
    break
for ky in allcounts.keys():
    allcounts[ky]["_N"] = float(len(allcounts[ky].keys()) - 1)

N = float(len(allcounts["_ALLCODES"].keys())) - 2.

del allcounts["_ALLCODES"]

entropies = []
for ky in allcounts.keys():
    entropies.append(computeEntropies((ky, allcounts[ky])))

likelihoodEntropy = {}
prior_entropy = {}

for x in entropies:
    code = x[0]
    entdict = x[1]
    priorNum = entdict["_"+code]
    prior_entropy[code] = priorNum
    del entdict["_"+code]
    likelihoodEntropy[code] = entdict

for code in prior_entropy:
    prior_entropy[code] = -np.log2(prior_entropy[code]/total_tweets)
###### end training
########################

```

```
###### testing
#########################
totals = {}
for code in codes:
    totals[code] = {}
    for thresh in thresholds:
        totals[code][str(thresh)] = {
            "TP": 0.,
            "FP": 0.,
            "FN": 0.,
            "TN": 0.
        }
for record in testingrecords:
    result = processTweet(record["tweet"])
    for code in codes:
        truth = record["codes"][code]
        for thresh in thresholds:
            st = str(thresh)
            if result["posteriors"][code]["1"] >= thresh:
                prediction = 1
            else:
                prediction = 0

            if truth:
                if prediction:
                    totals[code][st]["TP"] += 1.
                else:
                    totals[code][st]["FN"] += 1.
            else:
                if prediction:
                    totals[code][st]["FP"] += 1.
                else:
                    totals[code][st]["TN"] += 1.
for code in codes:
    for thresh in thresholds:
        st = str(thresh)
        try:
            totals[code][st]["TPR"] = totals[code][st]["TP"]/(totals[code][st]["TP"] + totals[code][st]["FN"])
        except:
            totals[code][st]["TPR"] = 0.
        try:
            totals[code][st]["PPV"] = totals[code][st]["TP"]/(totals[code][st]["TP"] + totals[code][st]["FP"])
        except:
            totals[code][st]["PPV"] = 0.
        try:
            totals[code][st]["F1"] = (2.*totals[code][st]["TPR"]*totals[code][st]["PPV"])/(totals[code][st]["TPR"]+totals[code][st]["PPV"])
        except:
            totals[code][st]["F1"] = 0.
        try:
            totals[code][st]["ACC"] = (totals[code][st]["TP"]+totals[code][st]["TN"])/\
                                      (totals[code][st]["TP"]+totals[code][st]["TN"]+ totals[code][st]["FP"]+totals[code][st]["FN"])
        except:
            totals[code][st]["ACC"] = 0.
    ## picking up here!
    st = max([(totals[code][str(thresh)]["F1"], str(thresh)) for thresh in thresholds], key=lambda x: x[0])[1]
    totals[code]["thresh"] = st
    print code, st, totals[code][st]["PPV"], totals[code][st]["TPR"], totals[code][st]["F1"], totals[code][st]["ACC"]
```

```
#######################
```

Code F Python code used to produce statistics from out-of-sample validation results.

1.6 Exploring social actions during New York City's climate change protest.

As part of this submission we also include a movie file which demonstrates how our trained adept Bayes classifier can track social actions as they unfold in real-time on the ground. This was accomplished by using the classifier to classify tweets during a multiple day climate change protest in New York City which began on September 21st, 2014. The movie file included as part of this submission is a Quicktime *.mov* file entitled **NYC-ClimateChangeProtests-Med.mov**.

The code and classified tweets used to produce this movie file and are included as part of this submission. The classified tweets used to create this movie file and S2 Fig 2 in the paper is **NY-CliMarch-classified.csv**.

The Python code which was used to create Figures 1, 2 and 3 in the paper is included below.

```
from pylab import *
import json, re, os
import matplotlib.pyplot as py
import matplotlib
from datetime import datetime
import smopy
from haversine import haversine
import numpy as np
from matplotlib.patches import Wedge
from geopy import geocoders

with open("~/projects/protest-project/data/tweets/timeseries/allTimes.json
    ", "r") as f:
    allTimes = json.loads(f.read())

with open("~/projects/protest-project/data/tweets/timeseries/timeseries/
    allActions/New York_timeseries.json", "r") as f:
    timeseries = json.loads(f.read())

thresholds = {
    "collective-force": 0.08,
    "collective-peace": 0.78,
    "singular-force": 0.92,
    "singular-peace": 0.85,
    "collective":0.79,
    "singular":0.87,
    "peace": 0.88,
    "force": 0.71,
    "action": 0.93
}

gn = geocoders.GeoNames(username = "username")

outdir = "~/projects/protest-project/data/tweets/timeseries/frames/
    processed/allActions/"
county = ["New York", "NY"]
handle = county[0]
figdir = "~/Documents/talks/APSA/images/"

colors = {
    "singular-peace": "black",
    "collective-peace": "blue",
    "collective-force": "red",
```

```
    "singular-force": "green",
}

codes = [
    "singular-peace",
    "collective-peace",
    "collective-force",
    "singular-force",
    "collective",
    "singular",
    "peace",
    "force",
    "action"
]
likelihoodEntropy = {}
for amb in codes:
    f = open("./binary/likelihood-entropy/Alameda_11-24_firstNight_recoded_stitched_allActions_"+amb+".json","r")
    for line in f:
        d = json.loads(line)
        for code in d:
            likelihoodEntropy[code] = d[code]

## for NY climate change protests
start = "2014-09-21-06-41"
end = "2014-09-27-09-50"
fignum = 1

for hour in timeseries["hours"]:
    fig = py.figure(fignum, figsize=(24, 16))
    fig.suptitle(", ".join(county)+": "+re.sub(":00","",str(datetime.strptime(hour,"%Y-%m-%d-%H").strftime("%m/%d/%Y %I:%M %p"))), fontsize=25, fontweight='bold')
    first_ax = fig.add_axes([0.0, 0.75, 1.0, 0.25])
    fignum += 1
    ylims = [0,0]
    xlims = [datetime.strptime(start,"%Y-%m-%d-%H-%M"), datetime.strptime(end,"%Y-%m-%d-%H-%M")]
    xpt = datetime.strptime(hour,"%Y-%m-%d-%H")
    if xpt < xlims[0] or xpt > xlims[1]:
        continue
    for action in colors.keys():
        x = []
        y = []
        for i in range(0,len(timeseries["hours"])):
            ypt = float(timeseries["posteriors"][action][i])
            xpt = datetime.strptime(timeseries["hours"][i],"%Y-%m-%d-%H")
            if xpt >= xlims[0] and xpt <= xlims[1]:
                if ypt > ylims[1]:
                    ylims[1] = ypt
            if timeseries["hours"][i] == hour:
                ix = i
            y.append(ypt)
            x.append(xpt)

        py.plot(x,y,color = colors[action], lw = 2, label = re.sub("-"," ",action))
        py.plot(x[ix],y[ix],color = colors[action], ms = 25, marker = ".")

    py.ylabel('Posterior presence of action', fontsize=25)
    py.xticks(fontsize = 20, rotation = 30)
    py.yticks(fontsize = 20)
    py.ylim(ylims)
    py.xlim(xlims)

    py.legend(loc='upper right', fontsize = 25)
```

```python

    ### now for the map ##########
    second_ax = fig.add_axes([0.0, 0.0, 0.6, 0.675])

    py.xticks(fontsize = 0)
    py.yticks(fontsize = 0)

    hoi = hour
    doi = re.sub("-\d\d$","",hoi)
    code = "collective-force"

    infile = outdir+handle+"_"+re.sub("-\d\d$","",hoi)+".json"

    f = open(infile, "r")
    for line in f:
        record = json.loads(line)
        if record["hour"] == hoi:
            frame = record
            break

    dims = (8,12)

    meanx, meany = gn.geocode(", ".join(county))[1]

    lon = [meany - 0.03, meany + 0.14]
    lat = [meanx - 0.03, meanx + 0.08]

    smomap = smopy.Map((lat[0],lon[0], lat[1], lon[1]), z=14)
    smomap.show_mpl(figsize=dims, ax = second_ax)

    pts = []
    pxpts = []

    for cluster in frame["clusters"]:
        xs, ys = smomap.to_pixels(np.asarray(cluster["center"][1]),np.asarray(cluster["center"][0]))
        if len(pts) < 2:
            pts.append([cluster["center"][1], cluster["center"][0]])
            pxpts.append([xs, ys])
        else:
            break
    try:
        hdist = haversine(pts[0], pts[1])
        pxdist = (((pxpts[0][0] - pxpts[1][0]) ** 2) + ((pxpts[0][1] - pxpts[1][1]) ** 2)) ** 0.5
        ppm = pxdist/(hdist*1000.)
    except:
        ppm = 1.
    fig = py.gcf()

    for cluster in frame["clusters"]:
        xs, ys = smomap.to_pixels(np.asarray(cluster["center"][1]),np.asarray(cluster["center"][0]))
        circ = py.Circle((xs,ys),ppm*(cluster["radius"]+25.),color='blue', alpha = 0.5)
        fig.gca().add_artist(circ)

        ## determine wedge angles!
        ang = 360.*(float(cluster["positives"][code])/float(cluster["size"]))
        if ang:
            sect = Wedge((xs,ys), ppm*(cluster["radius"]+25.), 0., ang, color="red", alpha=1.)
            fig.gca().add_artist(sect)

    mostIntense = 0
```

```
    intenseCluster = {}
    points = []
    for cluster in frame["clusters"]:
        intensity = float(cluster["positives"][code])
        for point in cluster["points"]:
            if point["posteriors"][code]['1'] > thresholds[code]:
                points.append(point)
        if intensity > mostIntense:
            intenseCluster = cluster
            mostIntense = intensity
    cluster = intenseCluster

    ### now for the shift ##############################
    third_ax = fig.add_axes([0.635, 0.0, 0.365, 0.675])

    counts = {}
    for point in points:
        for phrase in point["counts"]:
            counts.setdefault(phrase,0.)
            counts[phrase] += point["counts"][phrase]

    phrases = counts.keys()
    for phrase in phrases:
        if likelihoodEntropy[code].get(phrase,False) and \
        likelihoodEntropy["not."+code].get(phrase,False):
            counts[phrase] *= (likelihoodEntropy["not."+code][phrase] \
                               - likelihoodEntropy[code][phrase])
        elif likelihoodEntropy[code].get(phrase,False) and not \
        likelihoodEntropy["not."+code].get(phrase,False):
            counts[phrase] *= (likelihoodEntropy["not."+code]["_DEFAULT"] \
                               - likelihoodEntropy[code][phrase])
        elif not likelihoodEntropy[code].get(phrase,False) and \
        likelihoodEntropy["not."+code].get(phrase,False):
            counts[phrase] *= (likelihoodEntropy["not."+code][phrase] \
                               - likelihoodEntropy[code]["_DEFAULT"])
        else:
            del counts[phrase]
    bardata = sorted(zip(counts.keys(),counts.values()), key=lambda x: abs(x[1]), reverse=False)

    vals = [dat[1] for dat in bardata[len(bardata)-40:len(bardata)]]
    labs = [dat[0] for dat in bardata[len(bardata)-40:len(bardata)]]
    pos = arange(len(vals))+.5    # the bar centers on the y axis

    barh(pos,vals, align='center', color = "gray")
    for i in range(len(vals)):
        if vals[i] >= 0:
            text(vals[i]+.01*max(map(abs,vals)),pos[i]-.4, labs[i], fontsize=17)
        else:
            text(vals[i]-.01*max(map(abs,vals)),pos[i]-.4, labs[i], fontsize=17, ha = "right")
    ylabel('Rank by impact', fontsize=15)
    xlabel('Relative impact on classification', fontsize=15)
    xticks(fontsize = 15)
    yticks(fontsize = 15)
    ylim([-0.5,len(vals)+0.5])
    try:
        xlim([min(vals)-.15*max(map(abs,vals)) , max(vals)+.35*max(map(abs,vals))])
    except:
        continue

    labels = map(str, sorted(range(1,41), reverse=True))
    py.yticks(pos, labels, rotation='horizontal')
```

```

    plot_margin = 0.05

    x0, x1, y0, y1 = py.axis()
    py.axis((x0 - plot_margin*100,
             x1 + plot_margin*100,
             y0 - plot_margin*2,
             y1 + plot_margin*2))

    py.savefig(figdir+handle+'_3-panel_'+hoi+'.png', format = "png",
        bbox_inches='tight', pad_inches=0.25)
    py.close(fig)
```

Code G Version of the Python code used to create NYC climate march figures.

1.7 Copyright and Compliance

In collecting this data and producing this document, we have complied with all of the data collection terms and conditions stipulated by the *Associated Press (AP) Images Database* hosted by the University of California, Berkeley Library System and with all of the data collection terms and conditions stipulated as stipulated by *Twitter Inc.*.

All map images provided in the document were produced by the *Mapbox* package in *Python*: https://github.com/mapbox/mapbox-sdk-py. Tiles produced by the *Mapbox* package can be used under the Creative Commons 3.0 license (CC BY 3.0). More about this can be found here: http://maps.stamen.com.

Any questions or concerns regarding these matters should be directed to:

L. Jason Anastasopoulos
Email: ljanastas@uga.edu

References

1. Ricchiardi S. Covering the World: As US news organizations have backed away from foreign news coverage, the Associated Press' international report has become increasingly vital. American Journalism Review. 2007;29(6):32–40.

2. Rennie JD, Shih L, Teevan J, Karger DR, et al. Tackling the poor assumptions of naive bayes text classifiers. In: ICML. vol. 3. Washington DC); 2003. p. 616–623.

3. Williams JR, Bagrow JP, Reagan AJ, Alajajian SE, Danforth CM, Dodds PS. Selection models of language production support informed text partitioning: an intuitive and practical, bag-of phrases framework for text analysis. arXiv preprint arXiv:160107969. 2016;.
